# Chromatin arranges in chains of mesoscale domains with nanoscale functional topography independent of cohesin

**DOI:** 10.1126/sciadv.aba8811

**Published:** 2020-09-23

**Authors:** Ezequiel Miron, Roel Oldenkamp, Jill M. Brown, David M. S. Pinto, C. Shan Xu, Ana R. Faria, Haitham A. Shaban, James D. P. Rhodes, Cassandravictoria Innocent, Sara de Ornellas, Harald F. Hess, Veronica Buckle, Lothar Schermelleh

**Affiliations:** 1 Department of Biochemistry, University of Oxford, Oxford OX1 3QU, UK.; 2 MRC Weatherall Institute of Molecular Medicine, Haematology Unit, University of Oxford, Oxford OX3 9DS, UK.; 3 Micron Oxford Advanced Bioimaging Unit, University of Oxford, Oxford OX1 3QU, UK.; 4 Janelia Research Campus, Howard Hughes Medical Institute, Ashburn, VA 20147, USA.; 5 Spectroscopy Department, Physics Division, National Research Centre, 12622 Dokki, Cairo, Egypt.

## Abstract

Three-dimensional (3D) chromatin organization plays a key role in regulating mammalian genome function; however, many of its physical features at the single-cell level remain underexplored. Here, we use live- and fixed-cell 3D super-resolution and scanning electron microscopy to analyze structural and functional nuclear organization in somatic cells. We identify chains of interlinked ~200- to 300-nm-wide chromatin domains (CDs) composed of aggregated nucleosomes that can overlap with individual topologically associating domains and are distinct from a surrounding RNA-populated interchromatin compartment. High-content mapping uncovers confinement of cohesin and active histone modifications to surfaces and enrichment of repressive modifications toward the core of CDs in both hetero- and euchromatic regions. This nanoscale functional topography is temporarily relaxed in postreplicative chromatin but remarkably persists after ablation of cohesin. Our findings establish CDs as physical and functional modules of mesoscale genome organization.

## Teaser

Advanced FIB-SEM and 3D-SIM imaging uncover cohesin-independent chromatin arrangement into structural and functional modules.

## INTRODUCTION

The genome in mammalian cell nuclei is hierarchically organized at various scales correlating with diverse genomic functions (*1*, *2*). At the base pair to kilobase pair levels, DNA is wrapped around core histones to create nucleosomes (*3*). At the 100-Mb scale, entire chromosomes harbor distinct territories within the nucleus with transcriptionally active euchromatic and inactive heterochromatic segments tending to segregate into specific nuclear subregions (*4*). Local chromatin organization ranging from several kilobases to 100 Mb (“mesoscale”) remains poorly understood (*5*). Advances in next-generation sequencing–based chromosome conformation capturing methods (3C/Hi-C) have revealed partitioning into several hundred kilobases to a few megabase-sized topologically associating domains (TADs) (*6*, *7*). TADs are genomic segments with higher intra-TAD contacts compared to inter-TAD contacts. At higher levels of organization, TADs group into ~10- to 20-Mb genomic A and B compartments. A compartments denote deoxyribonuclease I–sensitive, transcriptionally active “open” chromatin, and B compartments denote transcriptionally repressed, “closed” chromatin (*8*). Compartments are generally correlated with euchromatin or heterochromatin, and nuclear interior or lamina/nucleolar contacts for A and B compartments, respectively (*9*).

A key regulator in TAD organization is CCCTC-binding factor (CTCF), which binds to convergently oriented recognition sequences that flank TADs and which define TAD boundaries at the linear genomic [one-dimensional (1D)] scale (*10*). Deletion of genomic TAD boundaries leads to aberrant transcriptional output (*11*). Co-occurrence of the ring-shaped cohesin complex at CTCF-binding sites suggest a regulatory role for cohesin in shaping TAD structures, possibly through a loop extrusion mechanism (*12*, *13*). Accordingly, loss of cohesin function leads to an erasure of TAD signatures on Hi-C interaction maps (*14*).

Whereas the linear genomic size of TADs is defined, their spatiotemporal organization is underexplored. Single-cell Hi-C and more recent microscopy-based approaches using fluorescence in situ hybridization (FISH) of TAD-based regions have observed a high degree of stochasticity and heterogeneity in chromatin conformations in fixed cells (*15*–*19*). Despite these advances in imaging single TADs, it is still unclear whether TADs always form a single physical entity, how they relate to adjacent nuclear compartments, and how active and silenced chromatin is distributed at TAD scales.

In silico simulations of chromatin as a melted polymer suggest that the charged properties of modified histone tails alter the compaction status of these chromatin domains (CDs) (*20*), which was more recently supported by single-molecule imaging experiments (*21*). This model explains the increase in the physical size of epigenetically active domains compared to epigenetically inactive domains (*22*). Decreased protein accessibility with increasing chromatin density may allow size differences between small transcription factors (TFs) and large nuclear macrocomplexes, such as the transcription machinery, to be exploited as a mechanism of transcriptional control (*23*).

Here, we use quantitative 3D super-resolution imaging of both fixed and live samples, complemented by 3D electron microscopy (EM) of cryo-preserved samples, to establish chromatin organization into TAD-sized irregularly shaped dense nucleosomal aggregations in the size range of ~200 to 300 nm. These mesoscale CDs are linked sequentially in a 3D curvilinear arrangement to form a distinct reticular network that is separated by a relatively wide RNA-filled interchromatin compartment (IC) space. We apply a novel high-throughput image analysis pipeline for 3D structured illumination microscopy (SIM) data to spatially map functional markers, such as structural proteins, histone modifications, and RNA polymerase II (RNAPII). We describe at physically relevant nanometer-scale distinct volumes or zones of genome function in the context of CDs. This organization is independent of cohesin, establishing CDs as functional and physical modules of mesoscale genome organization in mouse and human somatic cells.

## RESULTS

### Chromatin arranges in coherently moving submicrometer domains, distinct from an RNA-occupied IC

**Fig. 1:**
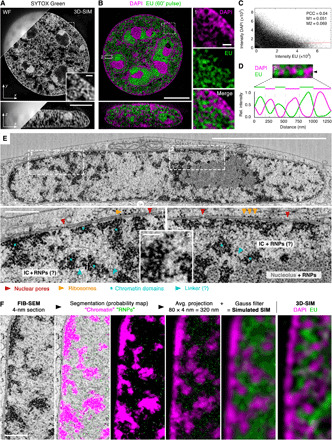
Chromatin folds into chains of mesoscale nucleosomal aggregates distinct from RNA-occupied interchromatin space. (**A**) Unlike conventional wide-field (WF, top left) imaging, SYTOX Green–stained chromatin in mouse C127 epithelial cells is resolved by 3D-SIM as a sponge-like reticular structure throughout the nuclear volume separated by unstained IC space. See also fig. S1. (**B**) IC juxtaposed to DAPI-stained chromatin is largely occupied by bulk RNA labeled with EU (60-min pulse). Right: Inset magnifications of region 1. (A and B) Scale bars, 5 and 0.5 μm (inset); lateral (top) and orthogonal (bottom) cross sections of 3D image stacks are shown. (**C**) Colocalization scatter plot confirms anticorrelation of DAPI and EU. PCC, Pearson correlation coefficient; M1/M2, Manders’ correlation coefficients DAPI/EU and EU/DAPI, respectively. (**D**) Line profile from a representative euchromatic region 2 in (B), highlighting mutually exclusive volumes occupied by DNA and RNA in the size range of 200 to 300 nm. (**E**) Single FIB-SEM image of a cryo-fixed HeLa cell milled in orthogonal direction and recorded with 4-nm pixel size. Scale bar, 5 μm. Bottom: Magnifications of boxed areas with postulated features annotated. Blue asterisks indicate 200- to 300-nm-wide aggregates of ~10-nm-sized dots, likely representing individual nucleosomes. Blue arrowheads indicate putative linker segments. Scale bars, 0.5 μm (insets 1 and 2) and 100 nm (inset 3). RNPs, ribonucleoproteins. (**F**) Columns 1 to 3: Machine learning–assisted segmentation of chromatin and putative RNPs. Columns 4 to 6: Average intensity projection of 80 consecutive segmented FIB-SEM sections covering a 320-nm *z* range, followed by 3D-Gauss filtering to match 3D-SIM’s spatial resolution, closely resembles the 3D-SIM micrograph at the same magnification. Scale bar, 0.5 μm. See also fig. S2.

The study of <1-Mb CD topologies in single cells in situ and in vivo has long been hindered, because they fall beyond the diffraction limit of light microscopy. Advances in super-resolution microscopy now put these structures within visual reach (*17*, *24*). 3D-SIM enables fast multicolor 3D acquisition of whole cells with eightfold higher volumetric resolution and strongly enhanced contrast compared to conventional fluorescence microscopy (*25*). Previous 3D-SIM studies resolved 4′,6-diamidino-2-phenylindole (DAPI)–stained chromatin in mammalian somatic cells as an intricate sponge-like structure, juxtaposed to an IC of no detectable chromatin that ends in channels leading to nuclear pores (Fig. 1A) (*26*–*28*). Using rigorously quality-controlled 3D-SIM (Materials and Methods and fig. S1A) in mouse (C127) and human (HeLa) somatic cells, we observe chromatin as a chain-like reticular structure of distinct nodes or CDs of variable diameters in the range of a few 100 nm, hereafter referred to as CD chains. DNA staining with either DAPI or SYTOX Green both show the same sponge-like pattern, with the exception of relative brighter chromocenter intensity in DAPI-stained mouse cell nuclei owing to its adenine-thymine (AT) base preference (Fig. 1A and fig. S1A). Control experiments in HeLa cells stably expressing green fluorescent protein (GFP) tagged histone H2B showed a near-complete colocalization of H2B-GFP and DNA staining (fig. S1B). Live-cell 3D-SIM imaging (Materials and Methods) confirms the presence of CD chains separated by IC space in vivo (fig. S1C) and unveiled a distinct dynamic behavior of these chromatin features over different observation periods and time intervals (fig. S2 and movies S1 to S3). Furthermore, quantitative flow field analysis (*29*) (Materials and Methods, fig. S2C, and movie S4) reveals localized spatially coherent chromatin motion, i.e., fields with same motion direction. These properties are consistent with mechanically coupled viscoelastic droplet-like domains (*29*, *30*) or “blobs.”

We next sought to better understand the content of the relatively wide IC space separating these domains. Considering RNA transcripts as a prime candidate, we performed an extended 5-ethynyluridine (EU) pulse labeling to detect all transcribed RNAs (*31*). We observed a notable enrichment of bulk RNA in the IC space including nucleoli and nuclear pore channels and almost mutual exclusion with DAPI-stained chromatin (Fig. 1, B and C). Line profiles through a representative euchromatic region of the nuclear interior highlight DNA domains in the size range of 200 to 300 nm alternating with the RNA-containing IC space of similar dimension (Fig. 1D).

The lateral resolution of our 3D-SIM imaging approach is limited to ~110 nm in the green emission range (fig. S1A). To test whether the observed nodal pattern of chromatin derives from structures at the molecular scale, we made use of high-resolution focused ion beam scanning EM (FIB-SEM). This approach allows imaging of entire nuclear volumes with <10-nm isotropic resolution (*32*). Cells grown on glass surface were cryo-fixed by high-pressure freezing followed by freeze substitution (FS) with osmium tetroxide and uranyl acetate, before resin embedding. This procedure ensures the best possible structural preservation and enhances the contrast of membranes as well as nucleoproteins (*33*, *34*). Assessment of FIB-SEM datasets of HeLa (G_1_ and G_2_) and U2-OS cell nuclei (Fig. 1E and fig. S3, A and B) allows identification of distinct CDs as densely packed aggregations of hundreds of highly contrasted ~10-nm-sized dots (likely nucleosomes) typically connected by linker filaments (Fig. 1E and movie S5). Chromatin at the nuclear periphery is seen as an inseparable melt, creating a homogeneous, almost continuous chromatin layer, except for nuclear pore complex (NPC) holes (fig. S3, C and D). The presence of >100-nm-sized higher-order domain features is of notable discordance with previous ChromEMT (*35*) results that show a more dispersed distribution of primarily 5- to 24-nm filaments by using more standard chemical fixation and sample preparation for transmission EM.

Using a machine learning approach (*36*), we next segmented CDs of aggregated nucleosomes distinct from nucleolar and IC regions (movie S6; Fig. 1F, columns 1 to 3; and fig. S3, D to F). The remaining sites marked solitary 10- to 20-nm-sized nuclear particles [likely RNA-protein complexes such as ribonucleoproteins (RNPs) or spliceosomes] within the IC. Projection and convolution with a Gaussian function of chromatin and RNP segmentation probability maps enabled us to generate a close mimic of 3D-SIM micrographs of DNA/RNA-stained cells (Fig. 1F, columns 4 to 6). Quantitative measurements of CD widths along the nuclear envelop yielded an average extent of ~200 ± 60 nm. Throughout the nuclear volume, the bulk of CDs have a dimension of 200 to 300 nm (fig. S3D). Smaller CDs (down to 100 nm) and larger CDs (up to >500 nm) are also apparent, but less frequent (movie S5).

We conclude that the bulk of nuclear RNA and DNA occupy mutually exclusive volumes. Both 3D-SIM and FIB-SEM independently identify chromatin as a convoluted, reticular structure composed of chains of submicrometer mesoscale CDs.

### CDs colocalize with TAD sequences and follow a curvilinear arrangement

**Fig. 2:**
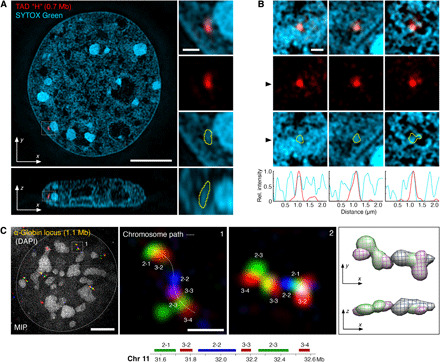
Spatial relationship between CD chain and TADs. (**A**) RASER-FISH detection of a 0.7-Mb X chromosomal TAD H (red; Hi-C map shown in fig. S4A) and SYTOX Green whole chromatin labeling (cyan) in a mouse C127 cell imaged by 3D-SIM. Right: Inset magnifications. Bottom: Orthogonal cross section. Yellow outlines of the FISH signal highlight ta close overlap with underlying CD chain nodes. (**B**) Further representative examples of TAD H FISH signals from separate cells outlined against chromatin. Bottom: Line profile of signal intensity for both TAD H (red) and SYTOX Green (cyan) calculated from a horizontal cross section at the black arrowhead. The spatial boundary of TAD H correlates strongly with CD boundaries. See also fig. S4 (A and B). (**C**) Multicolor RASER-FISH labeling of sequential TADs across the α-*globin* gene locus detected in mouse C127 cells. Maximum intensity projections (MIPs) of 3D-SIM micrographs show that the genomic order of the probes is preserved as a curvilinear chain of discrete CDs in space. Note that DAPI has only low affinity for single-stranded DNA and is only used here to indicate the nuclear outline. A second example region from a different cell is shown on the right as MIP and 3D rendering (with views from different angles). Scale bars, 5 and 0.5 μm (insets). See also fig. S4C.

Having established the arrangement of chromatin into spatially confined CDs, we used DNA FISH detection to place the observed 3D chromatin landscape in the context of physiological TADs as defined by Hi-C experiments. To avoid harsh denaturation and disruption of chromatin structures below ~1-Mb-size levels (*37*), we have implemented a nondenaturing FISH method, termed resolution after single-stranded exonuclease resection (RASER)–FISH (*38*, *39*). We applied RASER-FISH to study the topology of a previously characterized 0.7-Mb TAD “H” located on the mouse X chromosome (Fig. 2A and fig. S4A) (*19*). Quantitative analysis of 21 TAD H RASER-FISH signals revealed an average diameter of ~330 nm (fig. S4B). Although TADs in single cells are mostly globular, they are not uniform and some extend in at least one dimension to a maximum of ~500 nm (fig. S4B). The spatial boundary or rim of TAD FISH signals coincides with the rim of underlying CDs (Fig. 2, A and B). Furthermore, multicolor RASER-FISH of neighboring genomic domains spanning the α-*globin* locus revealed CDs arranged in a curvilinear path (Fig. 2C). Each genomic segment forms a discrete 3D nanodomain along a convoluted chain irrespective of their relative transcriptional activity (fig. S4C).

We conclude that chromatin in somatic interphase cells arranges as a convoluted curvilinear chain partitioned into discrete nonrigid shaped (globular or extended) CDs. These can harbor TADs and are separated from a DNA-depleted, RNA-occupied IC.

### Functional marker distribution reveals 3D zonation at CD scales

**Fig. 3:**
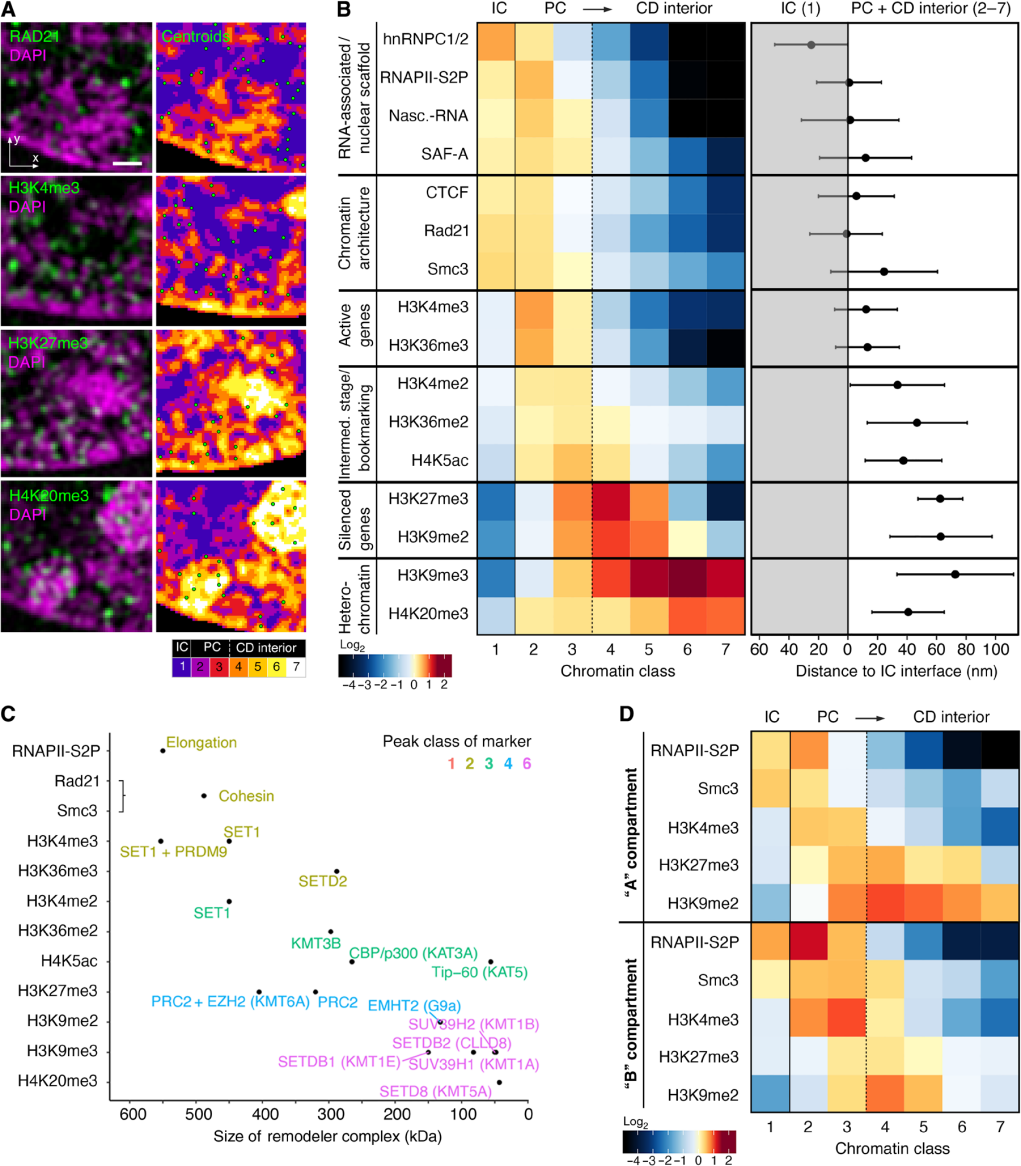
Functional marker distribution in somatic cells reveals nanoscale 3D zonation correlated with protein complex size. (**A**) Left: Representative subregions of single *z* planes of 3D-SIM image stacks of immunofluorescence (IF)–labeled mouse C127 cells for selected marker. Right: Centroid coordinates are depicted as green dots on top of segmented chromatin classes. G_1_ cell cycle stage was confirmed by the absence of EdU incorporation in combination with small nuclear volume. Scale bar, 0.5 μm. (**B**) Left: Heatmap of enrichment or depletion of IF signals relative to a random distribution (plotted in log_2_ fold change) for each chromatin class for each marker. Classes 2 and 3 are grouped as perichromatin (PC). Right: Distance to the IC interface for all markers. The mean distance and 95% confidence interval (CI) of the population are shown. At least 20 cells in two replicates were recorded for each marker, in total *n* = 433 cells. (**C**) Molecular weights of chromatin complexes corresponding to each marker. Scatterplot highlights an inverted correlation of size and preferential marker distribution (peak chromatin class color-coded). MW for cohesin was calculated from Smc1, Smc3, Rad21, and SA1/2. (**D**) Heatmap of relative IF signal distributions of representative markers in nuclear subvolumes harboring mainly heterochromatin characteristic of B compartments (the segmented perinuclear rim and chromocenter regions) or euchromatin characteristic of A compartments (the remaining volume). Both compartments show a relatively conserved zonation pattern despite the different composition of chromatin in either region (fig. S5D). Total number of cells *n* = 170.

The functional output of DNA is influenced by a broad repertoire of protein markers such as histone modifications, architectural proteins, transcription, replication, and chromatin remodeling enzymes. To understand how key functional markers map against the nuclear landscape at the nanoscale, we have devised a pipeline for automated high-content image analysis for the mining of spatial information from thousands of multicolor-labeled 3D-SIM nuclear volume datasets (for details, see fig. S5 and Materials and Methods). It first divides the chromatin signal into seven classes based on voxel intensity (*28*), which serve as a proxy for positioning other stainings relative to CD features (Fig. 3A and fig. S5E). The lowest-intensity region, class 1, denotes the DNA-depleted IC, classes 2 and 3 denote the outer rim or surfaces of CDs, previously termed perichromatin (PC) (*40*), and classes 4 to 7 constitute the more interior core region of the CDs, with classes 6 and 7 denoting primarily constitutive heterochromatin regions. We characterized the 3D epigenome topography using a wide range of markers (16 in total) associated with specific genome functions (nascent RNA), RNA-associated proteins, chromatin-associated proteins, and histone posttranslational modifications (PTMs; Fig. 3B and fig. S6A) in more than 400 mouse C127 cells in cell cycle stage G_1_ (table S1).

The RNA processing/interacting factors heterogeneous nuclear ribonucleoproteins C1/C2 (hnRNPC1/2), scaffold attachment factor A (SAF-A, also known as heterogeneous nuclear ribonucleoprotein U, hnRNPU), serine 2 phosphorylated RNAPII (RNAPII-S2P, marking actively elongating RNAPII), and nascent RNA transcripts are noted as enriched in class 1 (the IC) and the perichromatin classes 2 and 3, depleted in classes 4 and 5, and virtually absent in the highest classes 6 and 7 (Fig. 3B, top left). A recent study has shown the same pattern to be true for SC35, a protein involved in transcriptional splicing speckles (*41*). Those factors known to interact with chromatin, RNAPII-S2P, nascent RNA, and SAF-A, show a peak of enrichment in class 2, while the sole factor that only binds RNA, hnRNPC1/2, shows the strongest enrichment in class 1. Accordingly, average nearest-distance measurements to segmented chromatin surface result in a mean distance location of hnRNPC1/2 outside, while the other three markers center right at the borderline (Fig. 3B, top right). These results are in accordance with early immuno-EM observations that active transcription is confined to PC regions (*40*). They are also in line with the reported interaction of SAF-A with chromatin-associated RNAs (*42*) supporting the concept of a physical 3D interface between an RNA environment and less condensed outer CD fringes (Fig. 1B).

Unlike RNA-interacting proteins, the spatial distribution of segmented histone PTMs, which are inherently chromatin associated, show a marked depletion in the lowest-intensity class 1, further validating such annotated voxels as a true IC. We observe a biased enrichment for both H3K4me3, typically located at promoters of active genes, and H3K36me3, typically enriched along the body of transcribed genes (Fig. 3B, middle left), in the lowest chromatin classes 2 and 3 marking the PC, with peak enrichment in class 2 similar to RNAPII-S2P and nascent transcripts. These markers are rarely found in the higher-intensity classes 4 and 5, typically associated with the core of CDs, and are almost absent in the classes 6 and 7 (Fig. 3B, middle left). Their corresponding dimethylated forms (H3K4me2 and H3K36me2), as well as acetylated H4K5, which has been implicated in epigenetic bookmarking (*43*), also show a notable albeit less distinct enrichment in the lower-class range (peak in class 3) and depletion in higher classes.

In contrast, repressive histone PTMs generally show a broader distribution that is shifted toward the higher-intensity classes (Fig. 3B, bottom). The H3K27me3 mark, which is typically deposited along Polycomb-mediated silenced genes (*44*), shows enrichment in the interior classes (peaking at class 4). H3K9me3, a marker for transcriptionally inactive facultative and constitutive heterochromatin (*44*), is found enriched toward the CD interior and in heterochromatin classes (*3*–*7*) but is most abundant in the higher classes with a peak at class 6. H4K20me3, which has been implicated in silencing repetitive DNA (e.g., in chromocenters of mouse cells) and transposons (*45*), is most strongly enriched in classes 6 and 7, confirming these classes as constitutive heterochromatin. H3K9me2 shows a notably less shifted distribution than H3K9me3 peaking at the interior classes class 4 similarly to H3K27me3.

The observed differential enrichment of functional markers can also be considered in light of the specific enzyme complexes responsible for their deposition. There is a remarkable inverse correlation, with smaller complex sizes correlating to peak enrichment in higher chromatin classes of the corresponding marker (Fig. 3C). This supports the hypothesis that chromatin density acts as a higher-level regulator of genome function by hindering physical accessibility of larger complexes to substrate chromatin (*23*). Analyzing a subset of markers in human colon (HCT116) and cervical (HeLa) cancer cell lines in the G_1_ stage, we found highly similar differential spatial distributions, confirming the universal nature of the observed zonation (fig. S6, B and C).

It has been postulated that TADs may be wholly accessible or inaccessible depending on whether they form part of the A or B compartment, respectively, as denoted by Hi-C experiments. To test this, we subdivided the nuclear volume into lamina-associated chromatin and chromocenter regions, or the residual nuclear volume, as proxies for general B and A compartmentalization, respectively. We find the nanoscale zonation true for both euchromatic (“A”) regions and heterochromatic (“B”) regions (Fig. 3D and fig. S6D).Thus, both large-scale compartments harbor transcriptionally active and inactive sequences with nanoscale distributions, albeit at different ratios (*2*) (fig. S6E).

In conclusion, our high-content 3D super-resolution mapping approach highlights an ordered zonation of epigenetic markers, forming a nanoscale functional chromatin topography. The propensity of different functions (e.g., transcription silencing) to occupy different chromatin classes is independent of whether that chromatin is found in euchromatic or heterochromatic regions. Markers of active transcription and architectural proteins are confined to the PC (and CD linker regions), while silencing modifications are enriched toward the CD interior. The IC harbors most RNAs and RNA-interacting proteins. Last, we suggest that the packing of nucleosomes into mesoscale CDs may lead to the physical exclusion (or hindrance) of chromatin-associated complexes according to their size, thus providing a possible explanation for the ordered functional zonation as a consequence of physical accessibility (*23*).

### The local CD topography is temporarily relaxed during DNA replication

**Fig. 4:**
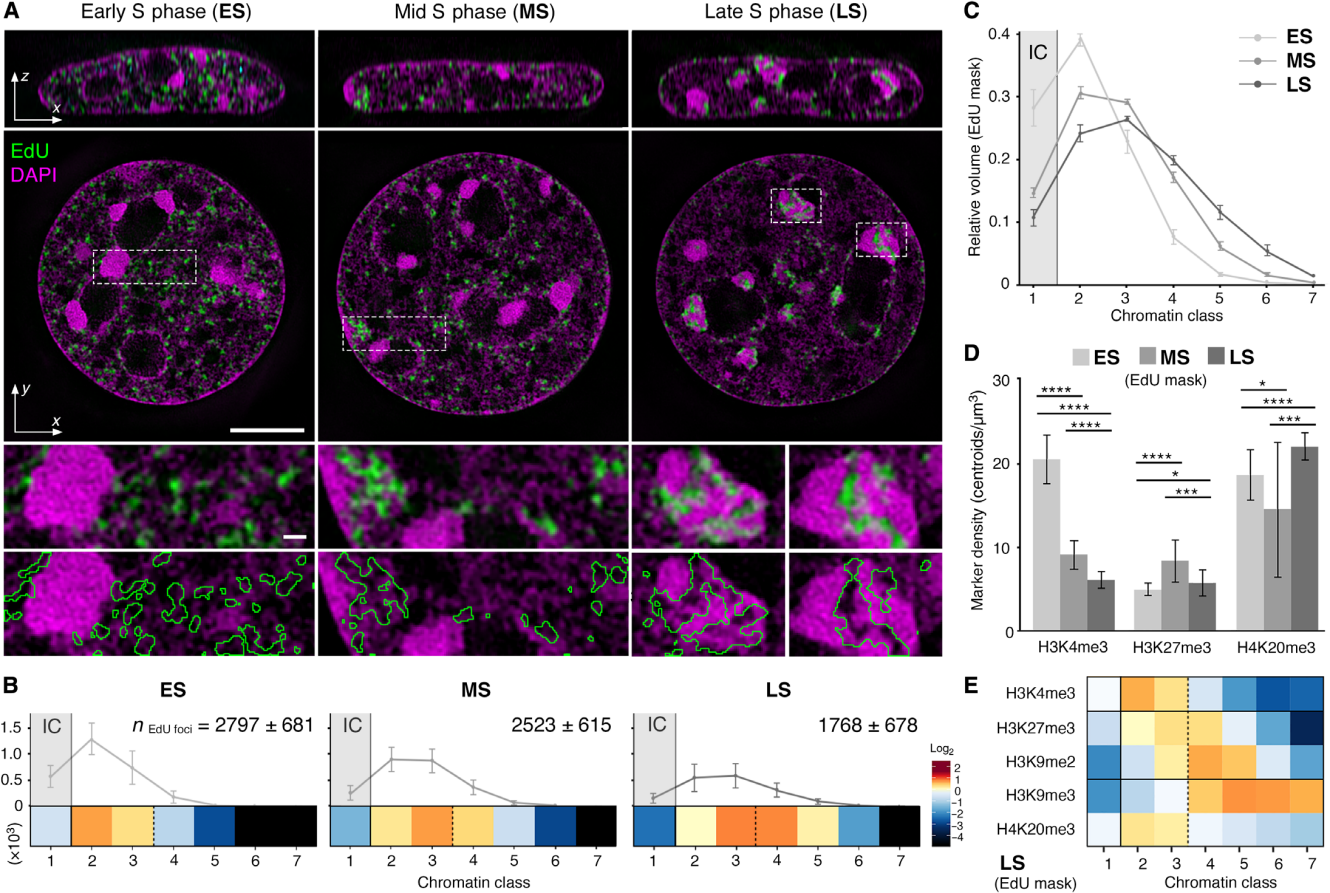
Effect of active replication on chromatin composition. (**A**) Orthogonal and lateral cross sections of representative 3D-SIM datasets showing the chromatin landscape with the different patterns of replication foci (green) during S-phase progression from early (ES), mid (MS), and late S-phase (LS) stages (left to right). Chromatin is stained with DAPI, and replication is labeled using 15 min of EdU incorporation. Local replication regions were masked on the basis of the EdU signal and outlined in green. Scale bars, 5 and 0.5 μm (insets). (**B**) Quantification of the absolute number of detected EdU foci in each of the different chromatin classes. The total number of EdU foci per cell is indicated as mean ± SD. Per S-phase stage, a heatmap of enrichment or depletion of EdU signals relative to a random distribution (plotted in log_2_ fold change) is depicted. (**C**) Proportion of chromatin classes (full nuclear volume segmented as shown in fig. S5E) within EdU submask volumes in different S-phase stage. The mean ratios ± SD are depicted. (**D**) Foci number per cubic micrometer of EdU submask volume for the depicted histone modification in each condition, per S-phase stage. Means ± SD are depicted. Number of cells: ES, 225; MS, 227; LS, 230. Significance values: *P ≤ 0.05; ***P ≤ 0.001; ****P ≤ 0.0001. (**E**) Heatmap of statistical distribution of selected marker in EdU-masked regions of late S-phase cells highlights a relaxation of repressive marker confinement to CD chain interior compared to G_1_ shown in Fig. 3B. See also fig. S7 for a complete list of marker distributions in all cell cycle stages.

DNA polymerases are part of large multienzyme complexes (replisomes) that assemble in a characteristic nuclear pattern of replication foci (*46*). These observations are in agreement with our finding of larger complexes being enriched at lower chromatin classes. However, DNA replication must eventually process the entire genome, irrespective of the activity state at any given locus. We hypothesize that every chromatin locus should, over time, be locally decompacted in the process of replication. To test this at the nanometer scale, we identified S-phase cells by short 5-ethynyl-2′-deoxyuridine (EdU) pulse labeling and grouped them into early, mid, and late S-phase stages. Subdividing the nucleus in this manner reveals a strong local decompaction at sites of ongoing replication as revealed by chromatin classification in the local vicinity of EdU foci (Fig. 4A). The disruption is confined to the local activity of the replication machinery, consistent with previous evidence (*21*), as no notable changes could be seen when analyzing the entire nuclear volume (fig. S7A). Local decompaction is markedly highlighted by tunnel-like voids becoming apparent in otherwise dense chromocenters in late S phase (Fig. 4A, right). This is in agreement with a recent study of DNA synthesis at constitutive heterochromatin (*47*). Accordingly, EdU-labeled replication foci show enrichment in the lower chromatin classes, irrespective of S-phase stage (Fig. 4B).

When quantifying chromatin classes in replicating subregions, we observed, as expected, a shift toward denser chromatin classes when progressing from early, to mid, to late S phase (Fig. 4C). Accordingly, the highest abundance of the active histone PTM H3K4me3 is in early S phase, as compared to the repressive PTM H3K27me3, which is most enriched in mid S phase (Fig. 4D and fig. S7B). Constitutive heterochromatin marks, in particular H4K20me3, become less enriched in denser chromatin classes during late S phase (Fig. 4E and fig. S7B) with the replication-driven opening of otherwise condensed chromatin. Our data show that active DNA replication can locally disrupt the physical organization of chromatin and, consequently, its functional zonation.

### Cohesin function is dispensable for 3D chromatin structure and functional zonation

**Fig. 5:**
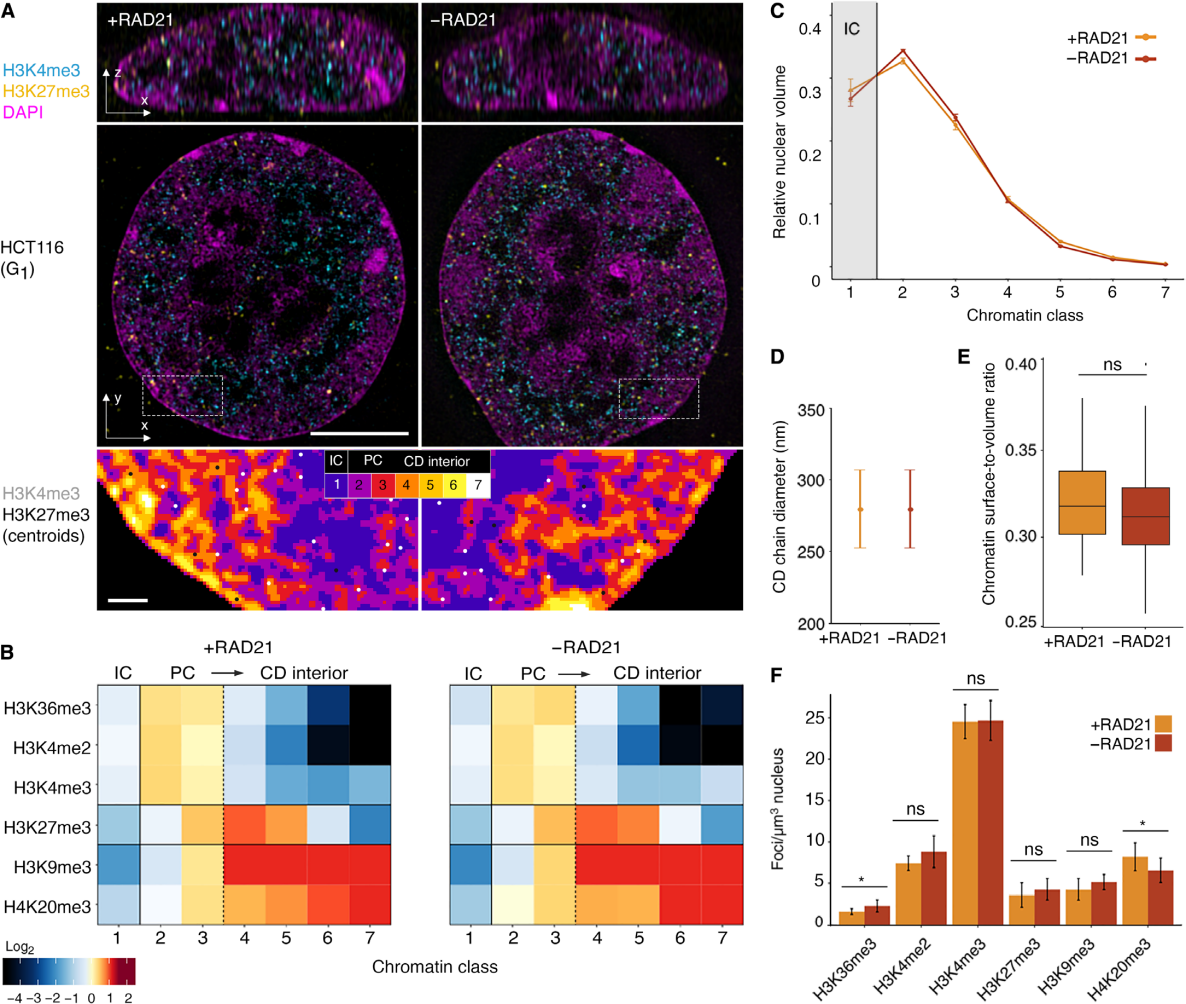
Epigenome zonation persists after cohesin ablation. (**A**) Representative single *z* planes of 3D-SIM images comparing the chromatin landscape in human HCT116 in the presence of cohesin and without cohesin. Control cells were only treated with auxin for 6 hours (+RAD21, left) or RAD21-ablated cells after 16-hour doxycycline and 6-hour auxin treatment (−RAD21, right). Insets show the segmented topography with the spatial distribution of H3K4me3 and H3K27me3 represented as white and black dots, respectively. Scale bars, 5 and 0.5 μm (insets). See also fig. S8 (A and B). (**B**) Heatmap of enrichment or depletion of IF signals from a representative subset of histone modifications. Respective profiles for individual markers with error bars show no significant change for any distribution; see also fig. S8C. (**C**) Proportion of nuclear volume for each class, showing no significant change after ablation of cohesin function (mean ± 95% CI). (**D** and **E**) Quantification of the CD dimensions [(D) ±95% CI; see Materials and Methods] and CD surface area–to–volume ratio [(E) ±SD)] shows no significant change between RAD21-positive or RAD21-depleted conditions. (**F**) Quantification of the mean number of foci detected for each condition shows almost no changes between RAD21-positive or RAD21-depleted conditions (±SD). Total number of cells: +RAD21, 80; −RAD21, 104. Significance values: *P ≤ 0.05; nonsignificant (ns) > 0.05.

While DNA polymerase is able to cause local rearrangements in S phase, other proteins are known to globally regulate chromatin structure throughout interphase. The cohesin complex is able to delineate the genomic boundaries of TADs (*13*) by a loop extrusion mechanism toward convergent CTCF-binding sites (*48*). In our analysis, the distributions of CTCF and two subunits of the cohesin complex, Smc3 and Rad21, were characterized by distinct enrichment in classes 1 and 2 and depletion in all higher classes (Fig. 3B, left). They also showed a distance profile centering at the 3D-segmented CD chain–IC interface (Fig. 3B, right). Cohesin degradation can erase TADs in Hi-C maps (*14*) but does not erase globular domains in FISH images (*17*). To address the role of cohesin on the global chromatin organization and the spatial zonation of the epigenome, we performed our analyses in human HCT116 cells with an auxin-inducible protease degradation system to ablate the RAD21 subunit of the cohesin complex (Fig. 5) (*49*). In addition, we added a C-terminal HaloTag allowing labeling with variable ligands to monitor the RAD21 expression level in each cell (fig. S8A). Upon addition of both doxycycline and auxin, RAD21 can be depleted with a half-life of 17 min (*49*) and completely ablated in 2 hours (fig. S8B). We hypothesized that if the epigenomic zonation were governed by sequence-specific CTCF-guided cohesin activity, then the 3D epigenome should be disrupted, while if this zonation were governed by nonsequence-specific biophysical constraints, then it should not change after cohesin depletion. We see no changes in the distribution of representative histone PTMs after 6 hours of cohesin depletion (Fig. 5, A and B, and fig. S8C).

Furthermore, quantitative analysis of HCT116 RAD21-mAID cells 6 hours after induction does not show any observable change in the CD chain structure (Fig. 5, C to E), nor are there changes in the number of functional histone PTMs on this landscape (Fig. 5F). The CD chain diameter and the ratio of segmented chromatin surface area relative to its volume do not change in the absence of cohesin, suggesting that cohesin is dispensable for these structures. This phenomenon has recently been observed to persist over longer periods of depletion (*41*).

Our findings demonstrate that loss of cohesin, and thereby of TADs (as defined by Hi-C), has no observable effect either on maintaining CD structures or on the epigenomic zonation relative to CDs in single somatic cells. We conclude that spatial functional zonation is regulated by the physics of the system independent of cohesin function and therefore not defined by specific sequences with cognate protein complex binding.

## DISCUSSION

**Fig. 6:**
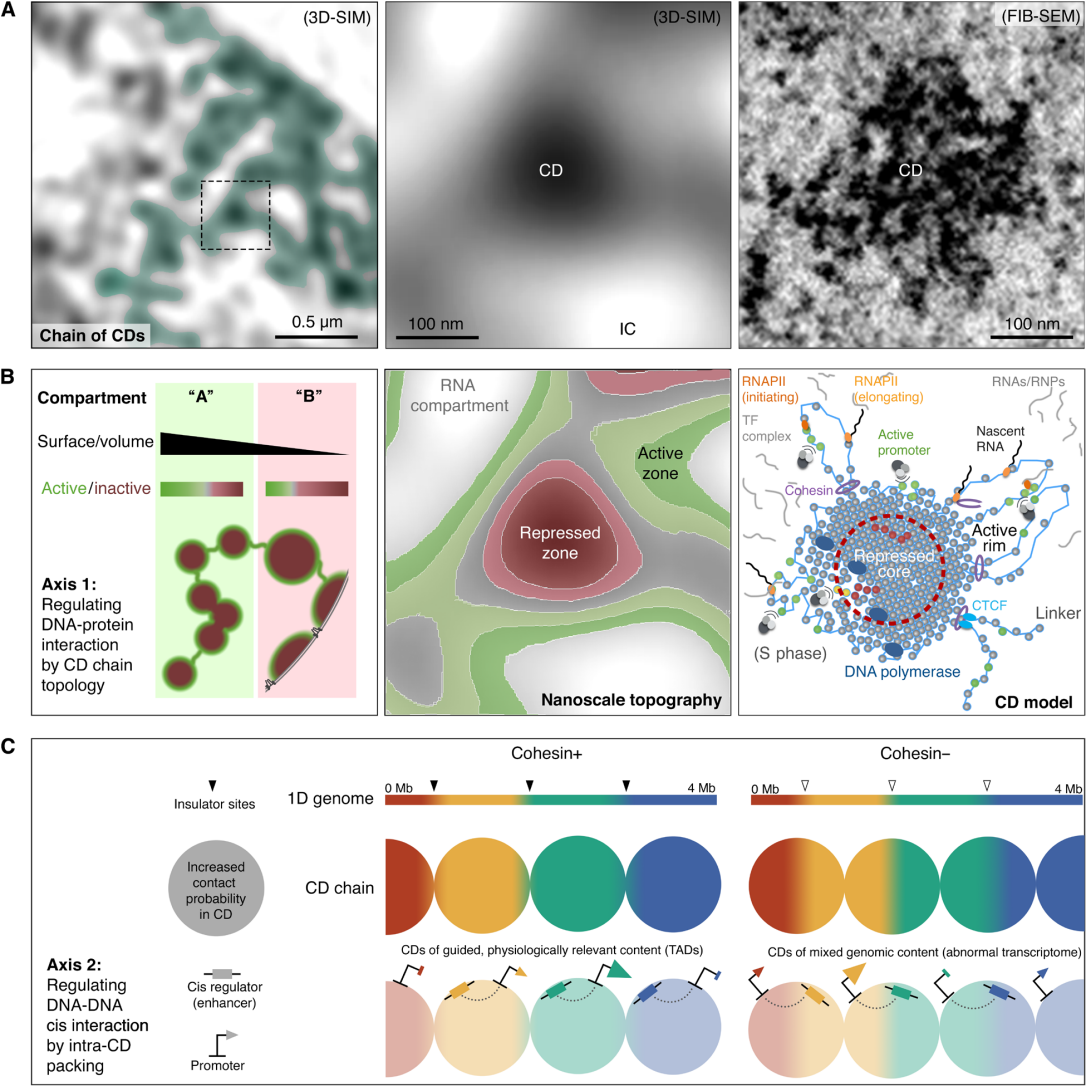
Model of genome organization into chains of CDs from the mesoscale to the nanoscale. (**A**) Left: 3D-SIM of DAPI-stained chromatin (inverted) with an overlaid representation of a putative curvilinear chain of CDs. Middle: Zoom-in to an individual CD, indicated by the dashed box in the left panel. Right: An equivalently sized CD imaged by FIB-SEM. (**B**) Functional zonation of CDs. Left: The CD chain’s surface area–to-volume ratio amenable to DNA-protein complex interactions provides one axis for regulating genome activity. Euchromatic A compartments and heterochromatic (often perinuclear) B compartments are characterized by CD chains with either an increased or decreased surface area–to-volume-ratio, respectively. Middle: Preferential zonation of nuclear functions at single CD level at the nanoscale. Right: Schematic CD model. Nucleosomes with active histone marks (green dots) and associated TF and RNA polymerase complexes are located at the outer fringes of CDs. In contrast, nucleosomes with repressive histone marks tend to relocate, either because of charge change or loss of complex binding, toward the CD core (red dashed circle), thereby becoming inaccessible to the transcriptional machinery. Local decompaction caused by DNA polymerase remodeling activity in S phase (dark blue ovals, left) may provide an opportunity for silenced loci to escape inactivation (yellow spots). (**C**) Supporting appropriate enhancer-promoter interactions is the second regulatory axis of CDs over genome function. Cohesin activity links the 3D structure to the correct 1D genomic context, guiding physiologically relevant interactions (left). CDs form also in the absence of cohesin but with less defined genomic boundaries, decreasing the probability of correct enhancer-promoter interactions (right).

By applying a rigorous quantitative high-content super-resolution 3D mapping approach of a wide range of functional markers, complemented by FIB-SEM, we establish that chromatin in somatic cells arranges in curvilinear chains of CDs of ~200- to 300-nm diameter, forming physical modules that subdivide the mammalian genome into functional volumes (Fig. 6A). CDs can harbor individual TADs in single cells and are spatially juxtaposed to and mutually excluded from an RNA-rich IC. The CD chain–IC organization features enrichment of diverse genome functions in different volumes, at the nanometer scale. Active transcription and architectural proteins are confined to the CD chain–IC interface, and silencing marks are enriched toward the core of the nucleosomal aggregates (Fig. 6B).

### CDs provide an axis of control over DNA-protein interactions

We demonstrate that nanoscale zonation occurs in both euchromatic and heterochromatic compartments of the nucleus, deviating from previous models of wholly active open versus inactive closed domains. On the basis of our data, we postulate that genome segments rich in active sites display a topology of multiple smaller or extended volumes and a higher surface area. Segments depleted in active sites, in contrast, form fewer more globular CDs or melts along the nuclear/nucleolar periphery with reduced surface areas (Fig. 6B, left). The large fraction of noncoding DNA present in the genome despite the associated energetic costs in its accumulation and replication has always been puzzling. However, the potential of using excess DNA in mesoscale CDs to regulate genome activity by means of accessibility may illuminate a raison d’être for much of the genome’s noncoding sequences.

CDs positioned at the nuclear envelope have less active interface due to the lamina blocking one flank. Observations from FIB-SEM reveal that CDs associated with the lamina, which are likely equivalent to lamina-associated domains (LADs) (*50*), also lack inter-CD separation by single-nucleosome linkers (a feature of CDs in the nuclear interior). Instead, they form a contiguous melt underneath the nuclear lamina. This conformation further reduces the available surface to just the nucleoplasmic flank (with the exception of channels reaching NPCs). Our model predicts this to favor silencing, which is true for LADs.

The described macroscale organization can be spatially inverted while maintaining normal physiological function in the nuclei of nocturnal mammalian retina cells (*51*). A model where regulation occurs at the nanometer scale is independent of the nuclear position of the CD at scales of tens of micrometers, as long as the local CD surface-to-volume ratio can be controlled. This provides a mechanism for how tissue-specific transcriptional programs can be established despite large variation in the spatial arrangements of macrocompartments between single cells (*52*), even in totally inverted architectures.

The ratio of active CD chain–IC interface area to CD chain volume should regulate genome activity by controlling the accessibility of nuclear machineries to their substrate, chromatin, although it is possible for some (e.g., DNA polymerase; Fig. 6B, right) to overcome these restrictions, reshaping the CD chain. We observe that zonation correlates with the size of chromatin-associated complexes, supporting in silico models. In previous modeling, accessibility of TFs is directly correlated to their molecular dimension, suggesting that binding of large TF complexes may “float as buoys” at domain surfaces, whereas smaller TFs are less affected by the steric hindrance of dense chromatin (*23*).

### CDs provide an axis of control over DNA-DNA interactions

We show cohesin activity, while essential for TADs in ensemble Hi-C, to be dispensable for maintaining an interphase chain of consecutive CDs. This is in line with a recent study by Bintu *et al*. (*17*) demonstrating that local 3D domains can form de novo in the absence of cohesin, i.e., in a sequence-unspecific manner. Elevated dynamics of nucleosomes was previously reported upon RAD21 depletion in HeLa cells by single-particle tracking (*21*), which hints at more subtle effects on a molecular-interaction level. At the mesoscale level, we conclude that CDs are not TADs, in that CDs represent the naive physical manifestation of chromatin derived primarily from its inherent polymer properties. Hence, CD formation is independent of sequence specificity and so cohesin independent.

We postulate that the role of cohesin-CTCF is to add a layer of sequence specificity on top of CDs exploiting the biophysically emergent structures to achieve physiologically relevant regulation between long-range cis elements (Fig. 6C, left). Without this cohesin-CTCF bridge between biology and physics, two outcomes become apparent. CDs would be composed of nonphysiologically relevant sequences, leading to an aberrant transcriptome (Fig. 6C, right). CD composition would also be highly variable from cell to cell, leading to an erasure of TAD signal from ensemble population Hi-C.

In contrast to cohesin, we have demonstrated that CDs and their nanoscale zonation can be locally and temporarily disrupted by active replication. The local relaxation of otherwise stable nucleosome aggregates may provide a window of opportunity for gene reactivation or for access by repair machineries to fix DNA damage from replication stress. In these scenarios, the adenosine 5′-triphosphate–driven activity of cohesin as a loop extruder or as a sister chromatid clamp may become critical, such as by stabilizing transient local higher-order chromatin structures that increase the fidelity of DNA double-strand repair (*39*).

### Possible mechanisms of CD formation

The concept of an IC permeating chromosome territories was introduced almost two decades ago (*4*). In such a model, the IC is a channel network that serves as transport highways for macromolecules to and from the nuclear pores. It was postulated that the PC region together with the IC constitute the active nuclear compartment (*28*, *53*). Here, by high-content analysis from 3D-SIM complemented by FIB-SEM, we show the IC to be a larger volume where a reticular network of CDs is found (fig. S5, B and C). Furthermore, transcriptional activity is enriched at a narrow CD chain–IC interface of less than 100 nm in width (Fig. 3B). Most of the IC volume is therefore not involved in active transcription.

Costaining for bulk RNA and DNA shows clear spatial exclusion of CDs from RNA-filled volumes. RNA transcripts, of which the vast majority are noncoding (*54*), are typically bound by intrinsically disordered proteins exerting multivalent interactions, known to have phase-separating properties (*55*), and many TFs are known to have intrinsically disordered domains facilitating phase separation (*56*). It is thus very likely that phase separation contributes to the observed CD architecture by establishing a separation of RNA/protein microcompartments, away from DNA-rich volumes.

Another driver of CD chain organization could be the default tendency of nucleosomes to self-interact into ever-larger collapsed polymer aggregates (“melts”) as previously suggested from single-nucleosome tracking and photoactivated localization microscopy experiments (*21*). CDs with coherent dynamics have been reported in recent live-cell and correlated super-resolution imaging of replication domains (*57*). In support of these observations, our FIB-SEM shows CDs as clearly separated entities, many of which are tethered to each other via apparent single-nucleosome-wide linker filaments (Fig. 1E, cyan arrowheads). These small linkers may offer mechanical coupling along the chain of CDs but remain flexible enough that each CD may move locally and independently from its neighbor. Being accessible, they also offer a site for the start of replication and transcriptional initiation.

These linkers also suggest that “stabilizing” forces promoting CD formation are counteracted by other forces. If this were not the case, then both phase separation or polymer melts would eventually lead to a complete separation of chromatin into a single large unit. We have shown that chromatin destabilization can result from replication in S phase. The destabilization by DNA polymerase may provide a window for TFs to access previously inaccessible genes (Fig. 6B, right). We suggest that transcription and cotranscriptional processes supported by repulsive electrostatic forces (exerted by acetylated histone tails) are likewise involved in interphase (e.g., at linker sites), tipping the local balance toward destabilization.

In conclusion, our novel nanoscale zonation model at the size of single physical CD entities allows for two independent axes of control. The first axis regulates DNA-RNA and DNA-protein interactions (Fig. 6B), and the second axis regulates DNA-DNA cis contacts (Fig. 6C). In the first axis, the control of genome function can be achieved through changes in the CD chain surface area. The shape of CDs varies the quantity of chromatin available as a substrate to epigenetic remodeler and transcription complexes at the CD-IC interphase. We show this to be true in euchromatic A and heterochromatic B compartments. In the second axis, CDs provide a structure that can be exploited by cohesin-based sequence-specific mechanisms for regulating interaction frequencies between long-range cis elements.

On the basis of these axes, our model predicts smaller domains with a larger surface-to-volume ratio in constantly replicating pluripotent stem cells and larger domains with a much-reduced surface-to-volume ratio in terminally differentiated or senescent cells that stop dividing. This would ensure either genome plasticity or structural “lock-in” and stabilization of specific functional states, respectively. Specific DNA-protein interactions could be tested in defined subsets of CDs by immunofluorescence (IF) relative to single-TAD RASER-FISH. Regulation of DNA-DNA interactions dependent on CD activity states could be monitored by live-cell tracking of multiple cis loci within or between individual CDs.

## MATERIALS AND METHODS

### Plasmids

pSpCas9(BB)−2A-Puro V2.0 targeting RAD21 [PX459 RAD21 (Hs)] was described previously (*58*). A poly glycine-serine linker, blasticidin resistance gene (BSD), GSG-P2A, mini auxin-inducible degron (mAID), and HaloTag were cloned between the Kpn I and Sal I site of a pUC19 vector by Gibson Assembly (New England Biolabs, catalog no. E2611). The pUC19 CT-BSD_GSG_P2A-mAID-HaloTag was cloned between two 1-kb homology arms at the 3′ of RAD21 to generate pUC19 RAD21 CT-BSD-GSG-P2A-mAID-HaloTag.

### Bacterial artificial chromosomes

The bacterial artificial chromosomes (BACs) used for FISH labeling of a single TAD were a gift from E. Heard (*19*). They correspond to X chromosome “TAD H” characterized between genomic loci 102325818 and 102998976 [673,158 base pairs (bp)]. The four BACs required to cover this region are 6-RP24-217I10 (102302874:102459532), 7-RP23-469A2 (102465652:102651023), 8-RP23-331 L13 (102648562:102834423), and 9-RP24-396 M14 (102804354:102976347), spanning a total length of 673,473 bp. The 315-bp difference between BAC coverage and TAD boundary calling is considered negligible over the 673-kb total (<0.05% error). BAC probes were directly labeled by nick translation using CF-594 (Biotium).

### Cell culture

Mouse mammary gland C127 and human cervical cancer HeLa H2B-GFP cells were grown in Dulbecco’s modified Eagle’s medium (DMEM) supplemented with 10% fetal bovine serum (FBS) and 1% penicillin and streptomycin. Human colon carcinoma HCT116 Tet-OsTIR1 cells were cultivated in McCoy’s 5A medium supplemented with 10% FBS, 2 mM l-glutamine, and 1% penicillin and streptomycin. Human lung fibroblast IMR-90 cells were grown in DMEM supplemented with 10% FBS and 1% penicillin and streptomycin. Cells were incubated at 37°C, 5% CO_2_ in a humidified incubator. At 90% confluence, cells were trypsinized with 0.05 to 0.25% trypsin in phosphate-buffered saline (PBS) or 1× trypsin replacement solution (TrypLE Express, Gibco) and passaged to a new culture dish at appropriate dilutions (1:5 to 1:12 every 2 to 3 days).

### Generation of inducible RAD21 degradation system

To generate a stable HCT116 Tet-OsTIR1 RAD21-mAID-Halo cell line, the pX459 RAD21 (Hs) plasmid and pUC19 RAD21 CT-BSD-GSG-P2A-mAID-HaloTag were transfected using FuGENE HD (Promega, catalog no. PRE2311). The single-guide RNA sequence used to generate the RAD21-mAID-Halo line was 5′-CCAAGGTTCCATATTATATA-3′. Two days after transfection, cells were grown in blasticidin-containing McCoy’s 5A medium (5 μg/ml). After 10 days, single colonies were transferred to 96-well plates, and homozygous clones were screened by polymerase chain reaction using the following primers: B2A-BSD: 5′-GCTCCTATTAGCCCTCCCACACATAACC-3′ (forward) and 5′-GATGTATAAAGGTTCCGGAGCCACCAAC-3′ (reverse); RAD21 (fragment 3): 5′-AGGGCTAATAAGGAGCTAGAAGCATTATAGC-3′ (forward) and 5′-TTGCATGCCTGCAGGGTTCCAAACCAGGAGTGTG-3 (reverse)′; RAD21 (fragment 1): 5′-AATTCGAGCTCGGTACTGAATGTCTGCAAAATGCCAAGC-3′ (forward) and 5′-CTTCTCCTTGCCGGAAATCTCGAGCGTC-3′ (reverse); mAID: 5′-TTTCCGGCAAGGAGAAGAGTGCTTGTC-3′ (forward) and 5′-CCGGAACCTTTATACATCCTCAATCGATTTTC-3′ (reverse); and genomic primer: 5′-AGCATGAGATTGGAGGGGA-3′ (forward) and 5′-GCCACTCAACATTATTACATGCA-3′ (reverse).

### EdU, EU, and BrUTP labeling and perturbation experiments

To identify cells in S-phase 10 μM EdU was added 15 to 30 min before fixation. G_1_ cells were identified by being negative for EdU pulse labeling and having a smaller nuclear size compared to G_2_ cells. For bulk RNA labeling, 1 mM EU was added 60 to 90 min before fixation. To label nascent RNA, a 10-min 5-bromouridine-5′-triphosphate (BrUTP) pulse labeling was performed by a scratch transcription labeling approach as previously described (*59*). For auxin-induced RAD21-degradation, HCT116 cells were cultured in the presence of doxycycline (5 μg/ml; Sigma-Aldrich, D9891) and 500 μM auxin (Sigma-Aldrich, catalog no. I5148) for 16 and 6 hours, respectively.

### RASER-FISH and probe labeling

RASER-FISH maintains nuclear fine-scale structure by replacing heat denaturation with Exonuclease III digestion of one of the two DNA strands after ultraviolet (UV)–induced generation of nicks and is suitable for high- and super-resolution imaging analysis (*38*). Briefly, cells were labeled overnight with a 3:1 BrdU:BrdC mix (5-bromo-2′-deoxyuridine:5-bromo-2′-deoxycytidine, respectively) at a final concentration of 10 μM. Cells were fixed in 2% formaldehyde for 15 min and permeabilized in 0.2% Triton X-100/PBS (v/v) for 10 min. Cells were incubated with DAPI (0.5 μg/ml in PBS), exposed to 254-nm wavelength UV light for 15 min, and then treated with Exonuclease III (New England Biolabs) at a final concentration of 5 U/μl at 37°C for 15 min. Labeled probes (100 ng each) were denatured in hybridization mix at 90°C for 5 min and preannealed at 37°C for 20 min. Coverslips were hybridized with prepared probes at 42°C.

### IF labeling

IF labeling was performed as described in detail (*60*, *61*). The antibodies used in this investigation are listed in table S2. One day before labeling, cells were grown on 22 mm by 22 mm #1.5H high-precision 170 ± 5 μm coverslips (Marienfeld Superior). HCT116 Tet-OsTIR1 RAD21-mAID-Halo cells were fluorescently tagged by incubating with 50 nM diAcFAM HaloTag ligand (Promega, catalog no. G8272) or 500 nM JF646 HaloTag ligand (gift from L. Lavis) for 30 min to monitor the expression level of individual cells. HCT116 cells were incubated in HaloTag-free medium for 30 min to remove residual ligand. Before fixation, cells were washed twice with PBS. Cells were fixed in 2% formaldehyde/PBS (Sigma-Aldrich, catalog no. F8775) for 10 min, washed with 0.02% Tween 20/PBS (PBST), and permeabilized in 0.1 to 0.5% Triton X-100/PBS. Coverslips were washed three times with PBST and incubated for 30 min in MAXblock (Active Motif, catalog no. 15252). Cells were stained with primary antibodies against the protein of interest, washed three times with PBST, and stained with fluorescently labeled secondary antibodies. After washing, cells were postfixed in 4% formaldehyde/PBS for 10 min and counterstained using DAPI (1 to 2 μg/ml) or SYTOX Green (1 μM) for 10 min. DAPI has a bias toward adenine and thymine over guanine and cytosine making AT-enriched satellite repeats near centromeric constitutive heterochromatin more intensively stained. This is particularly prominent in the chromocenters of mouse cells. This nonlinear correlation between signal intensity and DNA concentration meant that DAPI was replaced by SYTOX for all experiments possible. SYTOX has a weak affinity to bind RNA at high concentrations. Incubation with 1- to 10-U ribonuclease I/PBS before SYTOX counterstaining was performed as described (*59*). Coverslips were mounted in nonhardening VECTASHIELD (Vector Laboratories, catalog no. H-1000) and stored at 4°C.

### Super-resolution image acquisition, reconstruction, and quality control

3D-SIM images were acquired with a DeltaVision OMX V3 Blaze system (GE Healthcare) equipped with a 60×/1.42–numerical aperture PlanApo oil immersion objective (Olympus), pco.edge 5.5 scientific complementary metal-oxide-semiconductor (sCMOS) cameras (PCO), and 405-, 488-, 593-, and 640-nm lasers. For fixed-cell imaging, 3D image stacks were acquired over the whole cell volume in *z* and with 15 raw images per plane (five phases and three angles). Spherical aberration was minimized using immersion oil with refractive index (RI) 1.514 for sample acquisition. The raw data were computationally reconstructed with SoftWoRx 6.5.2 (GE Healthcare) using channel-specific optical transfer functions (OTFs) recorded using immersion oil with RI 1.512 and Wiener filter settings between 0.002 and 0.006 to generate 3D stacks of wavelength-dependent 110- to 130-nm lateral and ~320-nm axial resolution. Because of its broad emission spectrum, 405-nm excited DAPI staining was typically detected in the green emission range (and reconstructed with the “green” OTF) as this empirically improved the reconstruction quality. The system’s resolution was confirmed by measurements of monolayers of 100-nm-diameter yellow-green and red FluoSpheres (Thermo Fisher Scientific) (*61*). All SIM data were routinely and meticulously quality-controlled for effective resolution and absence of artifacts using SIMcheck (*62*), an open-source ImageJ plugin to assess SIM image quality via modulation contrast-to-noise ratio (MCNR), spherical aberration mismatch, reconstructed Fourier plot, and reconstructed intensity histogram values (*61*). Multichannel acquisitions were aligned in 3D using Chromagnon software (*63*) using 3D-SIM acquisitions of multicolor EdU-labeled C127 cells as colocalization reference (*60*). 3D-SIM imaging of DAPI or SYTOX Green–stained DNA typically provides a highly contrasted spatial representation of chromatin distribution with a lateral resolution of ~110 nm in lateral and ~320 nm in axial direction (fig. S1A).

For live-cell 3D-SIM, HeLa H2B-GFP cells were seeded in a 35-mm μ-Dish, high Glass Bottom (Ibidi) using phenol red–free DMEM supplemented with 10 μM Hepes. Live-cell imaging was performed at 37°C and 5% CO_2_ supply by using an objective heater and a stage top incubator. To improve the temporal resolution and reduce bleaching, only a central nuclear subregion of 7 *z* positions with a 125-nm interval was recorded (minimum required for 3D reconstruction), covering a total range of 0.75 μm and totaling 105 raw images (5 phases × 3 angles × 7 *z* positions) per time point. Using a 10-ms exposure time, frame rates of down to 0.5 Hz could be achieved, and typically, 12 to 18 time points could be recorded until the photon budget was exhausted because of photobleaching, with high-frequency noise (“hammer finish”) artifacts becoming overly prominent (*61*). The reconstructed *z* sections were processed in FIJI applying a maximum intensity *z* projection followed by Bleach Correction (histogram matching) and Linear Stack Alignment with SWIFT (affine transformation) to compensate for photobleaching and nuclear motion/deformation. Confining to a minimal stack height and subsequent projection further minimizes “*z* wrapping” artifacts described in (*61*). It is important to note that the above-described measures to enable live-cell 3D-SIM come at the expense of extended focus depth with reduced axial resolution (fig. S1C) and degrading reconstruction quality over time (movies S1 to S3).

### Quality validation

3D-SIM imaging and subsequent quantitative analyses is susceptible to artifacts (*61*). For instance, bulk labeling of densely packed chromatin inside mammalian nuclei of several-micrometer depth entails high levels of out-of-focus blur, which reduces the contrast of illumination stripe modulation and thus the ability to recover high-frequency (i.e., super-resolution) structural information. We therefore carefully assessed and optimized system performance as well as raw and reconstructed data quality using the SIMcheck ImageJ plugin (fig. S1A) (*62*). To exclude potential false-positive calls of nuclear marker signals, we used the MCNR map function of SIMcheck, which generates a metric of local stripe contrast in different regions of the raw data and directly correlates this with the level of high-frequency information content in the reconstructed data (*61*). Only immunofluorescent spot signals whose underlying MCNR values exceed a stringent quality threshold were considered, while localizations with low underlying MCNR values were discarded to exclude any SIM signal, which falls below reconstruction confidence. This criterion was applied to all datasets before feeding these to the analysis pipeline (fig. S9). We noted that most nuclear spots (between a few hundred to several thousand per nucleus, depending on the target and the antibody) were in the size range of a 3D-SIM resolution-limited point signal and that their intensities were similar to those few extranuclear spots originating from nonspecifically bound primary-secondary antibody complexes containing fewer than 10 dye molecules. We therefore conclude that nuclear signals predominantly represent the binding of a primary-secondary antibody complex to a single epitope.

### Focused ion beam scanning electron microscopy

Cryo-fixed HeLa (CCL-2) or U2-OS cells were prepared for FIB-SEM as described previously (*33*). Briefly, cells grown on sapphire coverslips (3-mm diameter and 0.05-mm thickness; Nanjing Co-Energy Optical Crystal Co.) were subjected to high-pressure freezing using a Compact 01 high-pressure freezer (Wohlwend), followed by FS under liquid N_2_ in FS medium (2% OsO_4_, 0.1% uranyl acetate, and 3% water in acetone) using an automated AFS2 machine (Leica Microsystems). Samples were washed immediately after FS three times in anhydrous acetone for a total of 10 min and embedded in Eponate 12. FIB-SEM imaging was performed using a customized Zeiss Gemini 500 (HeLa) or Zeiss Merlin (U2-OS) crossbeam system previously described (*32*). The Zeiss Capella FIB column was repositioned at a 90° angle to the SEM column. The block face was imaged by a 240-pA electron beam with 1.0-keV landing energy at 200 kHz (HeLa) or by a 2-nA electron beam with 1.2-keV landing energy at 500 kHz (U2-OS). The *x*-*y* pixel resolution was set at 4 nm (HeLa) and 8 nm (U2-OS). A focused Ga ion beam of 15 nA at 30 keV milled away ~3 nm (HeLa “G_1_”), ~5.6 nm (HeLa “G_2_”), and ~5 nm (U2-OS) of material from the block face for each section (thickness estimated on the basis of the average of SEM working distance and FIB milling voltage). The newly exposed surface was then imaged again. The milling/imaging cycle continued about once every 150 s for 1 month to capture an entire cell (50 μm by 8 μm by 68 μm) at a 4-nm voxel (HeLa) or every 40 s for 6 days to capture an entire cell (104 μm by 8 μm by 42 μm) at an 8-nm voxel (U2-OS). Each raw image stack was aligned and registered using a scale-invariant feature transform plugin through Fiji (*64*), and the U2-OS cell volume was further binned by a factor of 2 along the milling axis. A subvolume containing the nucleus was cropped from the whole cell stack for further processing and analysis. To correct for variations in the FIB milling thickness and ensure isotropic voxel sizes, a rescaling along the milling axis of 75% (HeLa G_1_), 140% (HeLa G_2_), and 125% (U2-OS) was applied (confirmed by the roundness of outer nuclear pore rings in horizontal planes). Machine learning–based segmentation of FIB-SEM data was performed using Ilastik 1.3.3 (*36*). 3D volume rendering after segmentation was performed with Imaris 9.2.

### ChaiN—a pipeline for high-content analysis of the 3D epigenome

ChaiN (Chain high-throughput analysis of the in situ Nucleome) describes a pipeline of scripts for the automated high-throughput analyses in this investigation (fig. S5). In brief, the counterstain/chromatin channel is used to generate (i) a nuclear mask and (ii) segment chromatin topography into seven intensity-based classes using an R script that is expanding on a hidden Markov model (*65*). The modal intensity value (composed of the large volume outside of the nucleus) is used to calibrate the relative intensity levels of each micrograph (due to unknown DNA dye incorporation and adjusted imaging conditions for optimized SIM imaging). Nucleoli were used as internal controls, as no signal should be detected in these volumes as expected. While this anchors the lower limit of the dynamic range, the upper limit is always relative to the maximum intensity value per micrograph, a limitation of the technique where cells of different conditions cannot be imaged together in the same micrograph for paired analysis. Nevertheless, on the basis of the output of ChaiN, we can quantitatively assess multiple parameters, such as the overall volume of the CD chain (PC + CD interior, combined classes 2 to 7; fig. S5C) versus the IC (class 1), the PC-IC 3D surface, and the surface-to-volume ratio. We note that the total number of discrete segmentation bins used in this analysis is arbitrary, with the sole criterion to group all nondetectable chromatin voxels as one class (class 1, IC).

The IF signals are thresholded by intensity using the Otsu algorithm, with all nonfoci voxels replaced by zeros. The complement of this image is segmented by a 3D watershed algorithm, and the 3D centroid coordinates are extracted from each segmented region’s center of mass after weighting by local pixel intensities. To filter out potential false-positive localizations, a local MCNR threshold is used to avoid potential artifacts skewing the statistical output (*61*, *62*). An MCNR mask was generated (using quality cutoff at MCN = 5). Thresholded images together with the MCNR mask were used as input for ChaiN to discard subquality voxels in the marker signal foci, reducing the effect of 3D-SIM artifacts on subsequent image analysis.

For each marker and condition, at least 20 3D datasets from two biological replicates were acquired, each with many hundreds to several thousands of annotated IF foci. Where relevant, significance testing was performed using a Student’s *t* test.

The quality-controlled and filtered centroid positions were then related to the chromatin-interchromatin volume in two ways. (i) The spot positions falling into each of the seven segmented classes were counted and normalized to the respective class size, and their relative enrichment (or depletion) was displayed in a heatmap on a log twofold scale. Indexing IF coordinates on the segmented chromatin yields their enrichment or depletion profiles at different chromatin densities normalized to the volumes of each chromatin class.

(ii) Segmentation of the 3D surface between class 1 and all other classes allows for a Euclidean distance calculation between the IF coordinates and the nearest CD chain surface voxel.

### Quantitative evaluation of chromatin dynamics

Spatial correlation dynamics of chromatin was estimated by using Dense Flow reConstruction and Correlation (DFCC) (*29*). Briefly, DFCC was applied to estimate the 2D apparent motion of two consecutive images in an image sequence (*N-1*); the direction and magnitude of flow fields of each pixel (size, 41 nm) were estimated for H2B-GFP over a 20-image series and with a time interval of 2 s. Then, spatial autocorrelation of scalar fields representing motion direction was calculated by fast Fourier transforms given by

$$r(\Delta x,\Delta y)=\frac{F^{-1}[F(\gamma )\cdot F^{*}(\gamma )]}{\langle \gamma \rangle \langle \gamma \rangle }$$

where F(·), $F^{-1}(\cdot )$, and $F^{*}(\cdot )$ are the Fourier transformation, the inverse Fourier transformation, and the complex conjugate of the Fourier transformation, respectively. Correlation was calculated as a function of space lag and fitted using the Whittle-Màtern model

$$r(\rho )=\frac{2^{1-\nu }}{\Gamma (\nu )}{\left(\frac{\rho }{\rho_{c}}\right)}^{\nu }K_{\nu }(\frac{\rho }{\rho_{c}})$$

where, ρ*_c_* and ν are the correlation length and a smoothness parameter, respectively. Correlation length and the smoothness parameter were derived from the regression and shown over the time lag. The parameters were averaged for each time interval over all accessible time points.

The Hi-D method was applied to estimate the biophysical properties of chromatin motion in living HeLa cells at single-pixel resolution (41 nm) (*66*). Mean square displacement (MSD) curves were plotted over time lags representing the trajectories of chromatin in the entire nucleus. Then, a Bayesian inference approach was applied to test the following five principle models for each MSD curve to classify types of motion. Fitting of the MSD curve derived from trajectories can be expressed for anomalous diffusion (α), free diffusion constant (D), directed motion (V), or a combination thereof. The value of each biophysical parameter extracted by this fitting is then mapped in a 2D color map, and the five principal models are shown as a colored map directly on the nucleus (fig. S3C). The five possible models for each MSD curve are

$$\text{MS}D_{D}(\tau )=4D\tau +o$$

$$\text{MS}D_{\text{DA}}(\tau )=4D\tau^{\alpha }+o$$

$$\text{MS}D_{V}(\tau )=v^{2}\tau^{2}+o$$

$$\text{MS}D_{\text{DV}}(\tau )=4D\tau +v^{2}\tau^{2}+o$$

$$\text{MS}D_{\text{DAV}}(\tau )=4D\tau^{\alpha }+v^{2}\tau^{2}+o$$

where D is the abbreviation of free diffusion model, DA is for anomalous diffusion model, V is for drift velocity model, DV is for free diffusion + drift model, DAV is for anomalous diffusion + drift model, α is anomalous diffusion exponent, τ is time interval, and *o* is a constant offset added to every model.

## Supplementary Material

aba8811_Movie_S1.mov

aba8811_SM.pdf

aba8811_Movie_S6.mov

aba8811_Movie_S5.mov

aba8811_Movie_S4.mov

aba8811_Movie_S2.mov

aba8811_Movie_S3.mov

## SUPPLEMENTARY MATERIALS

Supplementary material for this article is available at http://advances.sciencemag.org/cgi/content/full/6/39/eaba8811/DC1
