# Supplementary material for: Chromatin arranges in chains of mesoscale domains with nanoscale functional topography independent of cohesin
Source: Sci Adv. 2020 Sep 23;6(39):eaba8811. doi: 10.1126/sciadv.aba8811 (PMC7531892; doi:10.1126/sciadv.aba8811)
Supplement: aba8811_SM.pdf [file aba8811_SM.pdf]

Science Advances
AAAS

advances.sciencemag.org/cgi/content/full/6/39/eaba8811/DC1

Supplementary Materials for

Chromatin arranges in chains of mesoscale domains with nanoscale functional topography independent of cohesin

Ezequiel Miron, Roel Oldenkamp, Jill M. Brown, David M. S. Pinto, C. Shan Xu, Ana R. Faria, Haitham A. Shaban, James D. P. Rhodes, Cassandravictoria Innocent, Sara de Ornellas, Harald F. Hess, Veronica Buckle, Lothar Schermelleh*

*Corresponding author. Email: lothar.schermelleh@bioch.ox.ac.uk

Published 23 September 2020, *Sci. Adv.* **6**, eaba8811 (2020)
DOI: 10.1126/sciadv.aba8811

The PDF file includes:

Figs. S1 to S8
Tables S1 and S2
Legends for movies S1 to S6
References

Other Supplementary Material for this manuscript includes the following:

(available at advances.sciencemag.org/cgi/content/full/6/39/eaba8811/DC1)

Movies S1 to S6

Supplementary Figures

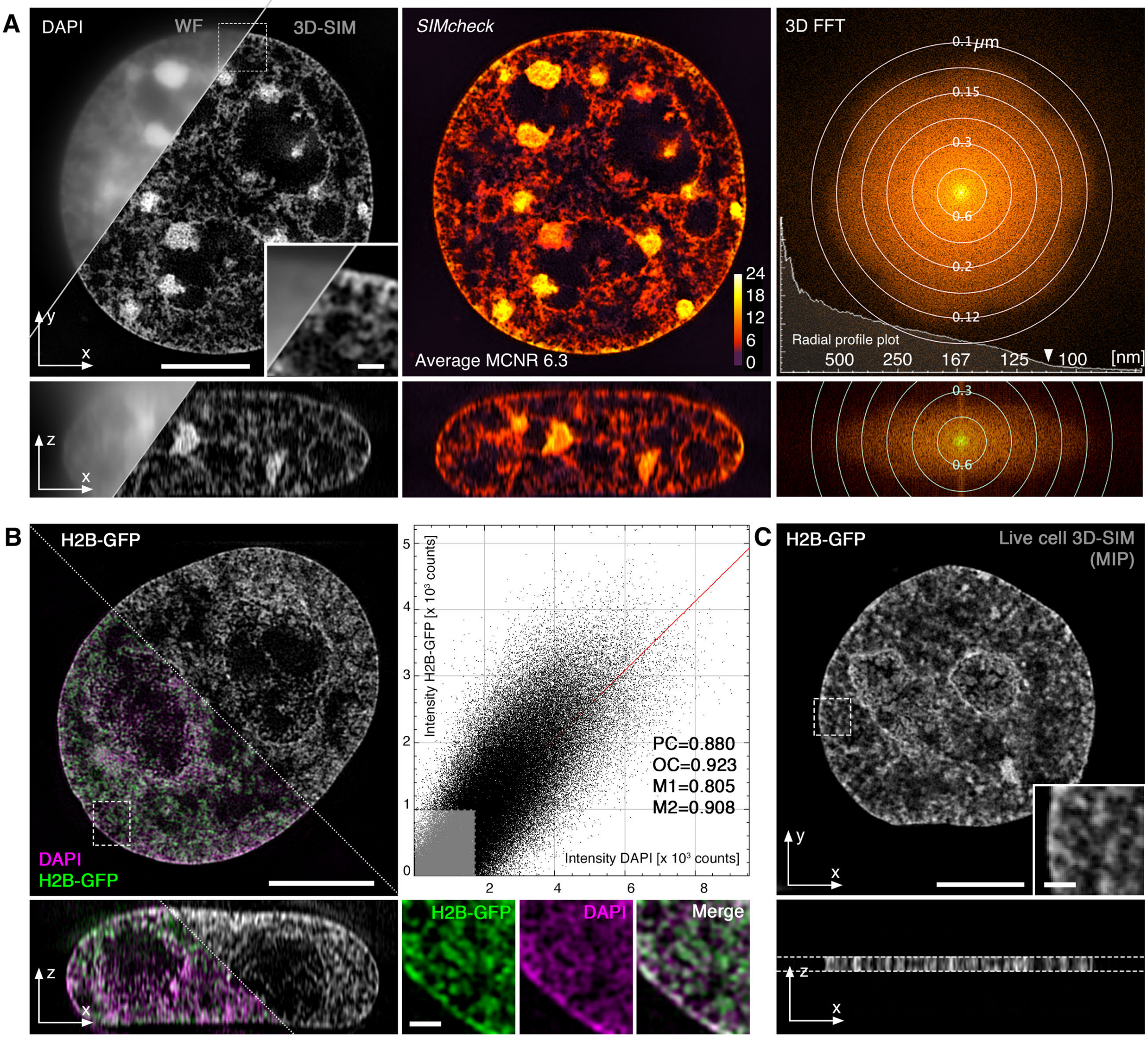

Fig. S1. Super-resolution imaging of chromatin. (**A**) 3D-SIM data quality control. Left panel: Comparison of widefield (WF) and corresponding 3D-SIM image of a mouse C127 cell nucleus stained with DAPI (excited with 405 nm, detected in green emission of 500-550 nm); lateral (top) and orthogonal (bottom) cross sections are displayed. Middle and right panels: corresponding quality control analysis using SIMcheck (*62*), showing a mapping of local stripe modulation contrast (Modulation Contrast to Noise Ratio, MCNR) on the reconstructed cross sections (middle), and the corresponding axial and lateral 3D fast Fourier transformation (FFT) plots with corresponding spatial resolution indicated by concentric rings and the radial profile plot shown superimposed (right). The effective lateral resolution is estimated from the inflection point (i.e. where the higher frequencies blend into the background frequency plateau), in this case at a frequency that is equivalent to ~110 nm spatial resolution (arrowhead). Similarly, the axial frequency distribution shown below almost reaches the equivalent of 0.3 μm z-resolution. (**B**) 3D-SIM image of DAPI-stained (magenta) fixed H2B-GFP (green) cells and corresponding colocalization analysis showing strong correlation of both signals. PC: Pearson correlation coefficient; OC: Overlap coefficient;

M1/M2: Manders' correlation coefficients DAPI vs. H2B-GFP and H2B-GFP vs. DAPI, respectively (using Otsu auto-threshold values indicated by grey box). (**C**) Live cell 3D-SIM image of histone H2B-GFP in stably expressing HeLa cell (corresponding to Movie S2). To increase temporal resolution and reduce bleaching, only 7 z-planes covering ~1 μm depth are acquired and, after processing, displayed as maximum intensity projection (MIP), at the expense of compromising axial resolution (orthogonal view). Note that chromatin features seemingly located within nucleoli are in fact peri-nucleolar heterochromatin domains located at the apical periphery captured by the extended sectioning depth.

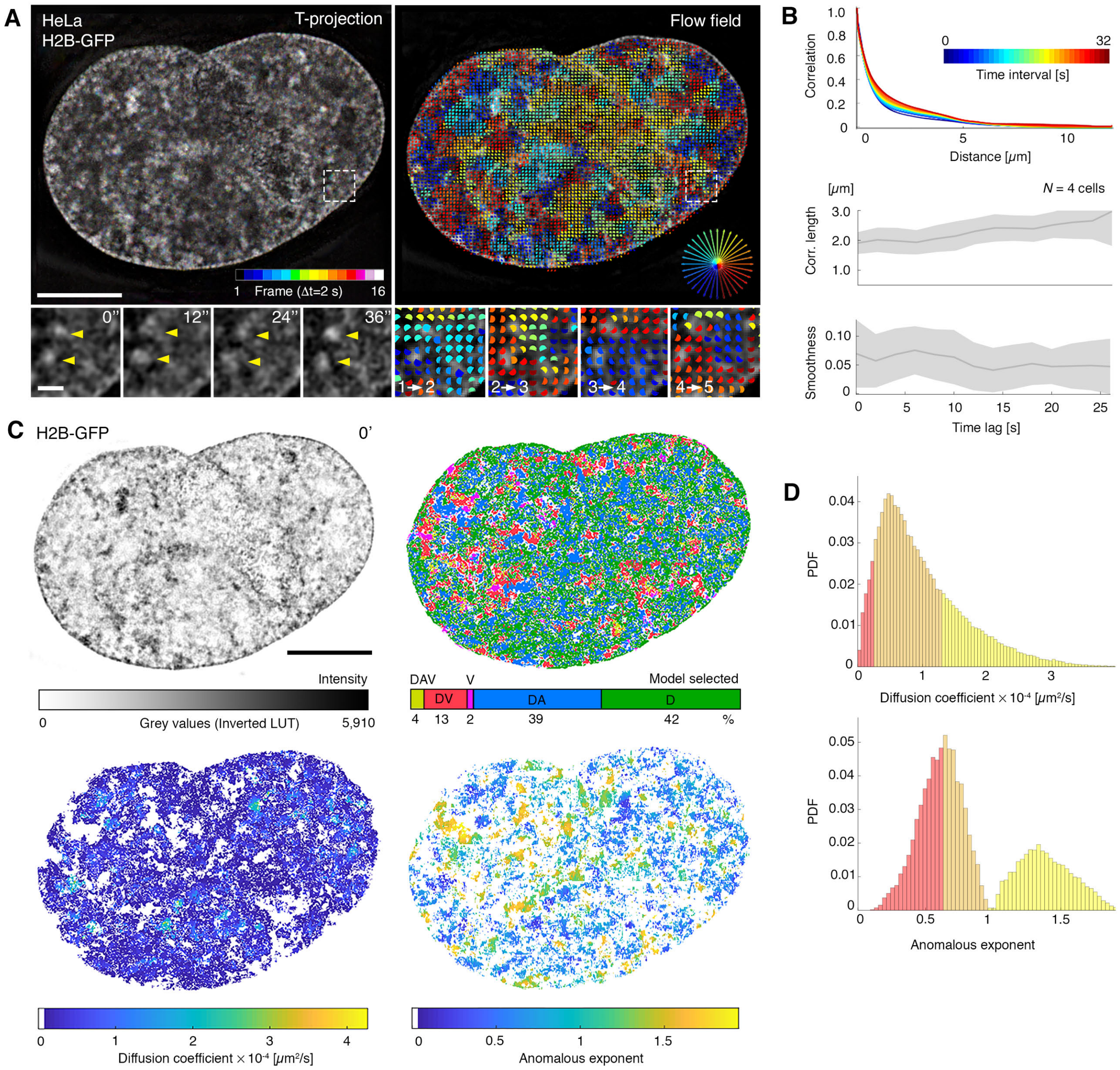

Fig. S2. Quantitative analysis of chromatin dynamics demonstrates the coherent movement of sub-micrometer domains and heterogeneity of chromatin motion with sub-diffusive and super-diffusive dynamic regimes. (**A**) Live cell 3D-SIM of human HeLa cells stably expressing histone H2B-GFP recorded with intervals of 2 s (see Movie S1 for entire time series; representative time lapse recordings with 3 s and 30 s are shown in Movies S2 and Movie S3, respectively). Left panel: Color-coded projection of 16 consecutive time-points. The seemingly absence of color information indicates relative stability of higher order structures over time. Inset magnifications show selected time-points of the boxed region as a maximum projection of 7 z-sections covering ~1 μm depth. Inset magnifications of selected individual time-points showing coherently moving domains (arrow heads). Right panel: Overlay of flow field indicating motion direction for the first time interval. Fields are color-coded according to the direction of the displacement (Movie S4). Insets show analyses for the first 4 intervals with a time lag of 1 frame (Δ=2 s). Scale bars: 5 μm and 0.5 μm (insets). (**B**) Correlation function (top) calculated from dataset shown in panel A as a function of distance at every accessible time interval (Δt is color coded from short to long time

intervals, from blue to red, respectively) within the image series, each of them fitted to the Whittle–Matérn model. Correlation length (middle) and the smoothness parameter (bottom) are calculated over time for both direction and magnitude of flow fields. Results show a correlation length of ~ 2 to 3 μm for time intervals between 2 s and >20 s, indicating a change in correlation length of 0.4-0.5 μm between adjacent coherently moving domains (middle), for directional correlation of flow fields. This is accompanied by smoother transitions between these adjacent domains (bottom). (**C**) Quantitative analysis of motion fields is applied using Bayesian model selection to define the types of diffusion processes acting on the chromatin fiber (top left) at the local and global scales. The spatial distribution of the selected models for each pixel is shown as a color map (top right), where D: Free diffusion model; DA: Anomalous diffusion model; V: Drift velocity model; DV: Free diffusion + drift model; DAV Anomalous diffusion + drift model. The mapping of the diffusion coefficient and the anomalous exponent in the lower panels highlight the heterogeneity of chromatin motion. (**D**) Histograms of diffusion coefficient and anomalous exponent values (panel C, lower right) plotted against the probability density function (PDF) after deconvolution using a general mixture model. For diffusion coefficient distributions, three mobility population groups, slow, intermediate and fast (shown as red, orange and yellow, respectively) are identified. The anomalous exponent distribution reveals two sub-diffusive populations (red and orange) and a third population with a super-diffusive regime (yellow).

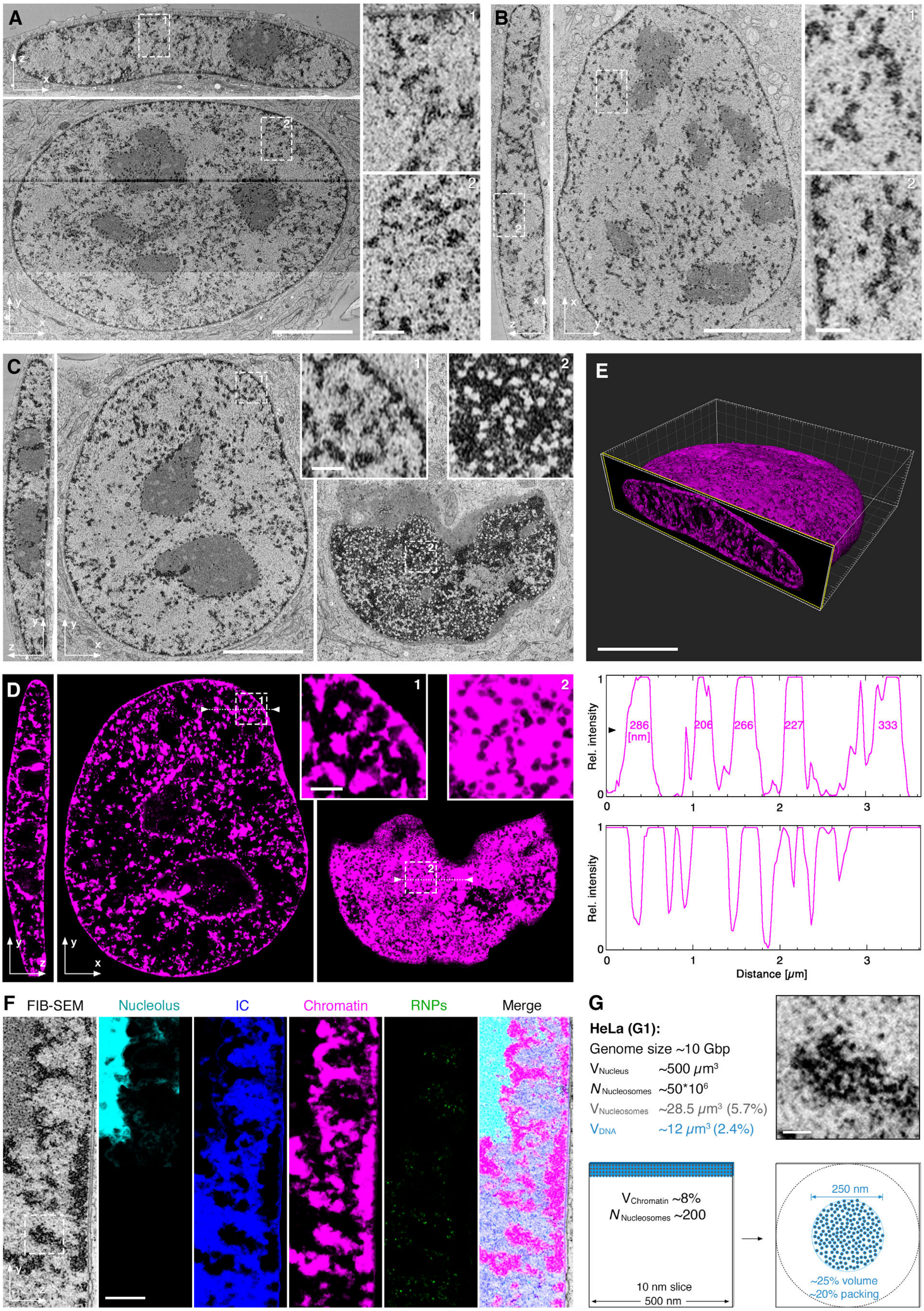
A
B
C
D
E
F
G
Rel. intensity
286 [nm]
206
266
227
333
Distance [μm]
FIB-SEM
Nucleolus
IC
Chromatin
RNPs
Merge
HeLa (G1):
Genome size ~10 Gbp
V Nucleus ~500 μm³
N Nucleosomes ~50*10⁶
V Nucleosomes ~28.5 μm³ (5.7%)
V DNA ~12 μm³ (2.4%)
V Chromatin ~8%
N Nucleosomes ~200
10 nm slice
500 nm
250 nm
~25% volume
~20% packing

Fig. S3. Chains of CDs imaged with FIB-SEM. (**A**) Single FIB-SEM sections in orthogonal (x-z; milling plane) and lateral (x-y) direction (relative to the growth surface) of a cryo-fixed HeLa (G2) cell (stage determined by centriole maturation and nuclear volume). The sample was stained with osmium and uranyl acetate under cryo-conditions before freeze substitution, resin embedding and FIB-SEM imaging. The 3D dataset, acquired with 4 nm pixel size and ~5.6 nm milling thickness, has been downscaled to 20 x 20 x 20 nm^3 isotropic voxel size for convenience. (**B**) Single orthogonal and lateral FIB-SEM sections of a cryo-fixed U2-OS cell acquired with 8 nm pixel size and ~10 nm milling thickness, downscaled to 20 x 20 x 20 nm^3 isotropic voxel size. Similar chromatin features can be observed for both HeLa and U2-OS nuclei (inset magnifications). (**C**) Orthogonal and lateral FIB-SEM sections of the HeLa (G1) cell dataset shown in Fig. 1E, F, downscaled to 20 x 20 x 20 nm^3 voxel size. (**D**) Machine-learning-assisted segmentation of chromatin shown in magenta for the same data shown in panel C. The dimension of CDs is typically in the size range of 200-300 nm (line profile 1, top right), with lamina associated CDs being no larger than CDs in the interior. Lamina associated CDs show no discernable linkers but instead form a continuous "melt" in an axis parallel to the nuclear lamina (right inset; line profile 2, bottom right, dips in intensity correspond to nuclear pores). (**E**) 3D volume rendering of chromatin segmentation from the dataset in panel D. (**F**) Machine-learning-assisted segmentation of a high-resolution (4 nm pixel size) sub-volume of the FIB-SEM dataset shown in Fig. 1E, F. Classification of 4 different states within the nuclear volume: the nucleolus (cyan), the interchromatin compartment (IC, blue), chromatin (magenta), and individual high contrast particles, likely RNPs (green). (**G**) Estimated average number, volume ratio and packing density of nucleosomes in a volume of 500 x 500 x 10 nm in a HeLa G1 cell given the genome size, the estimated number of nucleosomes, the volume of nucleosomes, and the volume of DNA. Distribution of the estimated number of 200 nucleosomes in an idealized CD of 250 nm diameter (bottom right) in comparison with a typical CD (boxed region in panel F) displayed at the same size scale (top right). Scale bars for all panels: 5 μm and 0.5 μm (for insets and magnified regions).

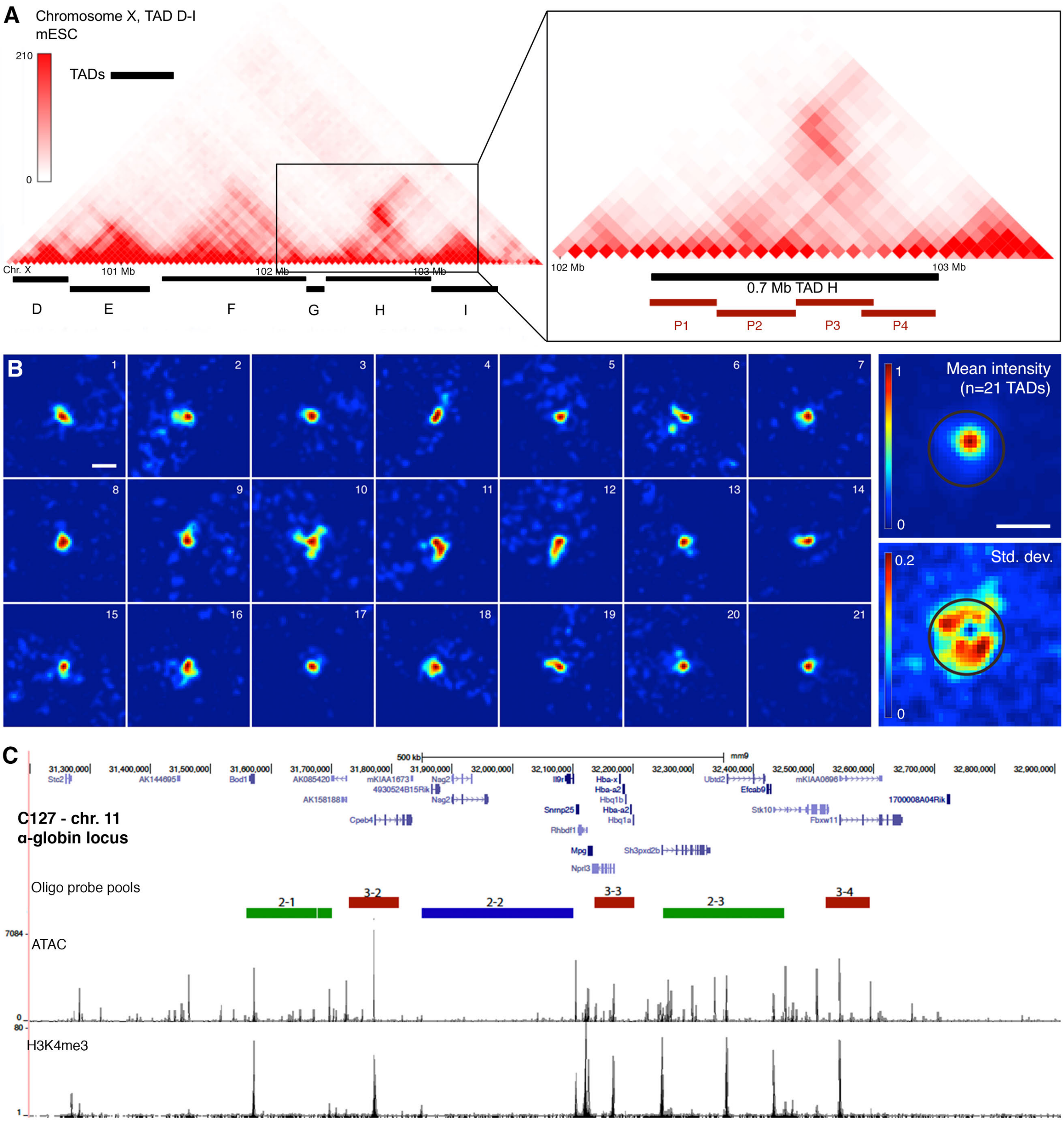

Fig. S4. RASER-FISH for imaging chromatin conformation. (**A**) Hi-C heatmap from mouse ESCs (*67*) showing the interaction frequency around the 700kb TAD probe (probe H, red bars), (*19*) used in this study for RASER-FISH experiments. (**B**) Left: collection of TAD H signals on the X chromosome. Heatmaps of single z-planes are shown. The displayed z-planes contain the brightest pixel of the TAD z-stack. Signal intensity was normalized to the brightest pixel. Top right panel: quantification of the mean intensity of 21 TADs. For each TAD, the z-plane containing the brightest pixel was selected, centered with respect to this pixel and normalized to its signal intensity (top right). The standard deviation (SD, bottom right) describes the degree of variation in maximum elongation of TADs, indicated with a diameter of ~500 nm. Scale bar: 0.5 μm. (**C**) Chromatin features in C127 cells showing sequential domains (see Fig. 2C) characterized by the presence or absence of H3K4me3 ChIP-Seq and ATAC-Seq peaks.

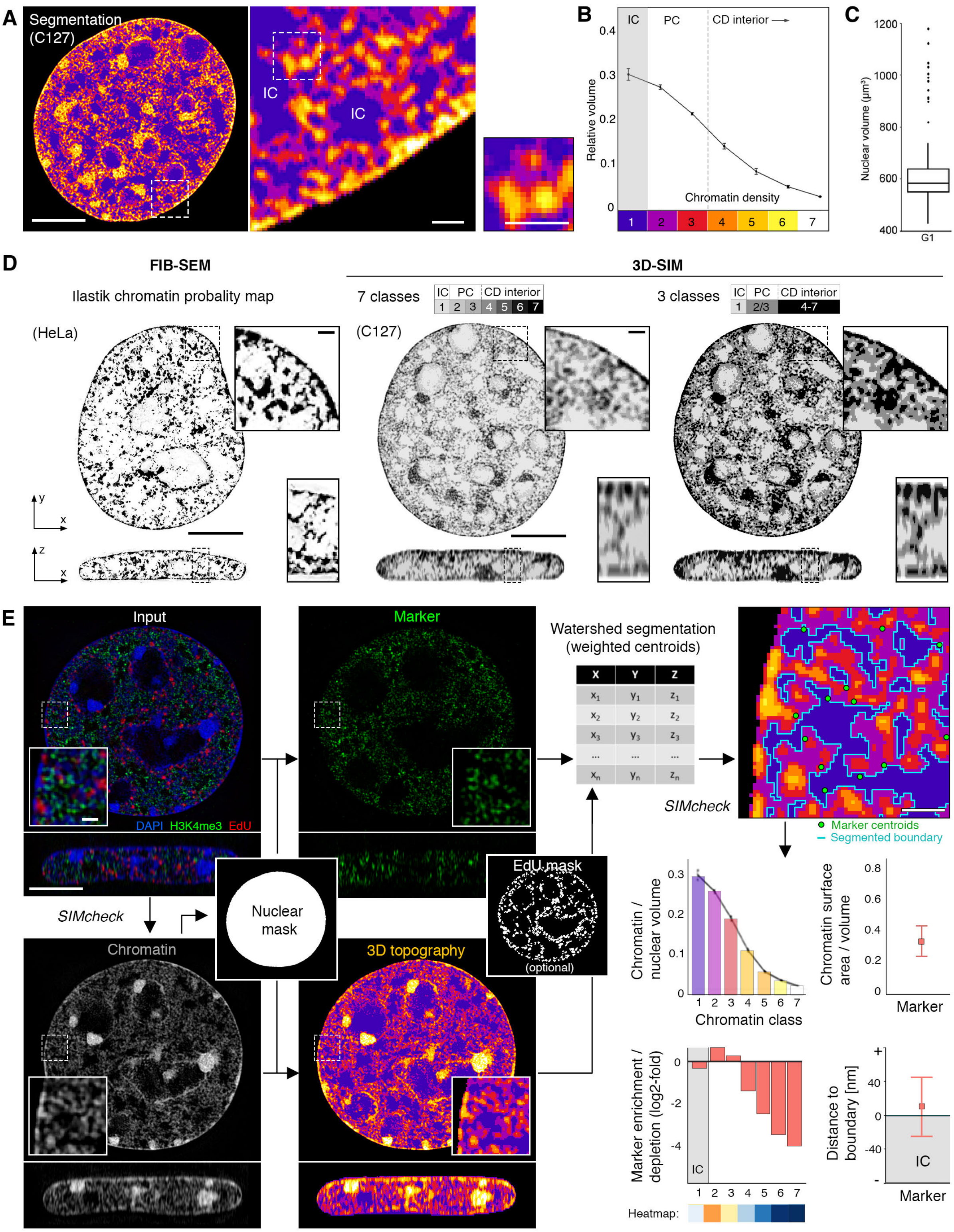

Fig. S5. Systematic analysis of the 3D epigenome. (**A**) Segmentation of chromatin staining into 7 intensity classes (Methods) at three different serial magnifications. (**B**) Quantification of relative class volumes from interchromatin compartment (IC, class 1), perichromatin (PC, class 2-3) and core regions of CD chains (CD interior, classes 4-7) shows a decrease of nuclear volume fractions for higher classes. The IC and PC together account for ~75% of nuclear volume from SIM data. Error bars indicate 95% CI. Relative

volume as a proportion of the total volume within the nuclear mask. (**C**) Box plot of nuclear volumes in C127 G1 cells from all segmented nuclear voxels. $N = 433$ cells (panels B and C). (**D**) Comparison of segmentation methods for FIB-SEM (left) and 3D-SIM (center, right) data. Lateral (top) and orthogonal cross section (bottom) are shown. Grouping the 7-class segmentation (center) into class 1, classes 2-3 and classes 4-7 (right) most closely resembles the FIB-SEM segmentation (left), validating the clustering of classes into 3 main states: IC, PC and CD interior (as in panel B).) Note that the anisotropic resolution of 3D-SIM along the z-direction, highlighted in the orthogonal sections (bottom), skews the comparison with isotropic FIB-SEM data. (**E**) Schematic representation of the ChaiN analysis workflow. Inputs for the workflow are multi-channel 3D-SIM datasets that have been pre-processed, quality-controlled, thresholded and aligned. First, a nucleus mask is generated based on the chromatin channel. Secondly, within this mask, marker spots are segmented by intensity and their weighted centroid positions are determined. Simultaneously, the chromatin channel is segmented into 7 intensity classes as detailed above to describe the 3D topography of chromatin. Optionally, the segmented chromatin can be sub-queried by a further round of masking using a binary mask derived from a biological region of interest, e.g., A vs. B compartments (see Fig. 3D) or EdU signal for postreplicative chromatin (see Fig. 4C-E). Finally, metrics from both marker and chromatin channels are used to describe the 3D nucleome, such as the nuclear proportion of each chromatin class, the chromatin surface-to-volume ratio, the marker enrichment or depletion in each chromatin class, or the mean distance (± 95% CI) of markers to the segmented chromatin-interchromatin edge. Analysis is performed by linking scripts written in R and Octave. Scale bars for all panels: 5 μm and 0.5 μm (insets).

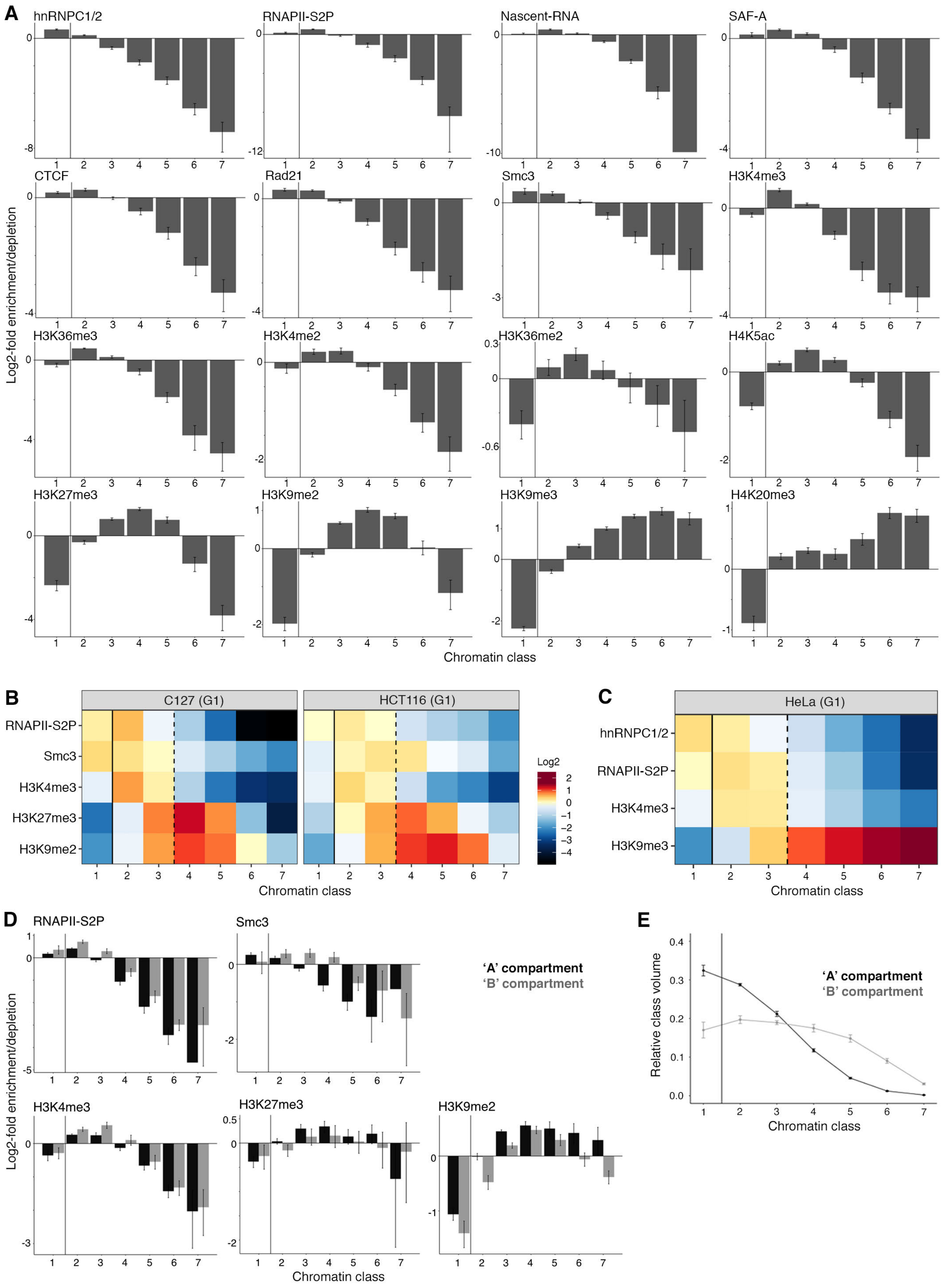
A
hnRNPC1/2
RNAPII-S2P
Nascent-RNA
SAF-A
CTCF
Rad21
Smc3
H3K4me3
H3K36me3
H3K4me2
H3K36me2
H4K5ac
H3K27me3
H3K9me2
H3K9me3
H4K20me3
Log2-fold enrichment/depletion
Chromatin class
B
C127 (G1)
HCT116 (G1)
RNAPII-S2P
Smc3
H3K4me3
H3K27me3
H3K9me2
Log2
Chromatin class
C
HeLa (G1)
hnRNPC1/2
RNAPII-S2P
H3K4me3
H3K9me3
Chromatin class
D
RNAPII-S2P
Smc3
'A' compartment
'B' compartment
H3K4me3
H3K27me3
H3K9me2
Log2-fold enrichment/depletion
Chromatin class
E
Relative class volume
'A' compartment
'B' compartment
Chromatin class

Fig. S6. Functional marker distributions in whole mouse and human cell lines and macro-compartments. (**A**) Bar plots of the log-2 fold enrichment or depletion of each analyzed marker in each of the segmented chromatin classes, in mouse C127 G1 cells, relative to chromatin class volume. Numbers of cells for each marker is detailed in Table S1. (**B**-**C**) Representative selections of markers in mouse C127 cells show the same zonation patterns with the same antibodies in human HCT116 colon carcinoma cells (panel B) and HeLa cells (panel C), suggesting chromatin accessibility of PC (classes 2-3) vs CD interior (classes 4-7) as a universal regulator of epigenetic zonation. Number of cells: C127 = 170, HCT116 = 70, HeLa = 41. (**D**) Bar plots of the over/under representation of representative markers in each of the segmented G1 macro-scale A/B chromatin compartments, showing a relatively conserved zonation pattern between active and repressive markers. Plots show average log-2 value, error bars indicate 95% confidence interval. Number of cells = 170. (**E**) Composition of chromatin classes in either A or B regions. Plot shows relative class volume, error bars indicate 95% confidence interval. Number of cells = 170.

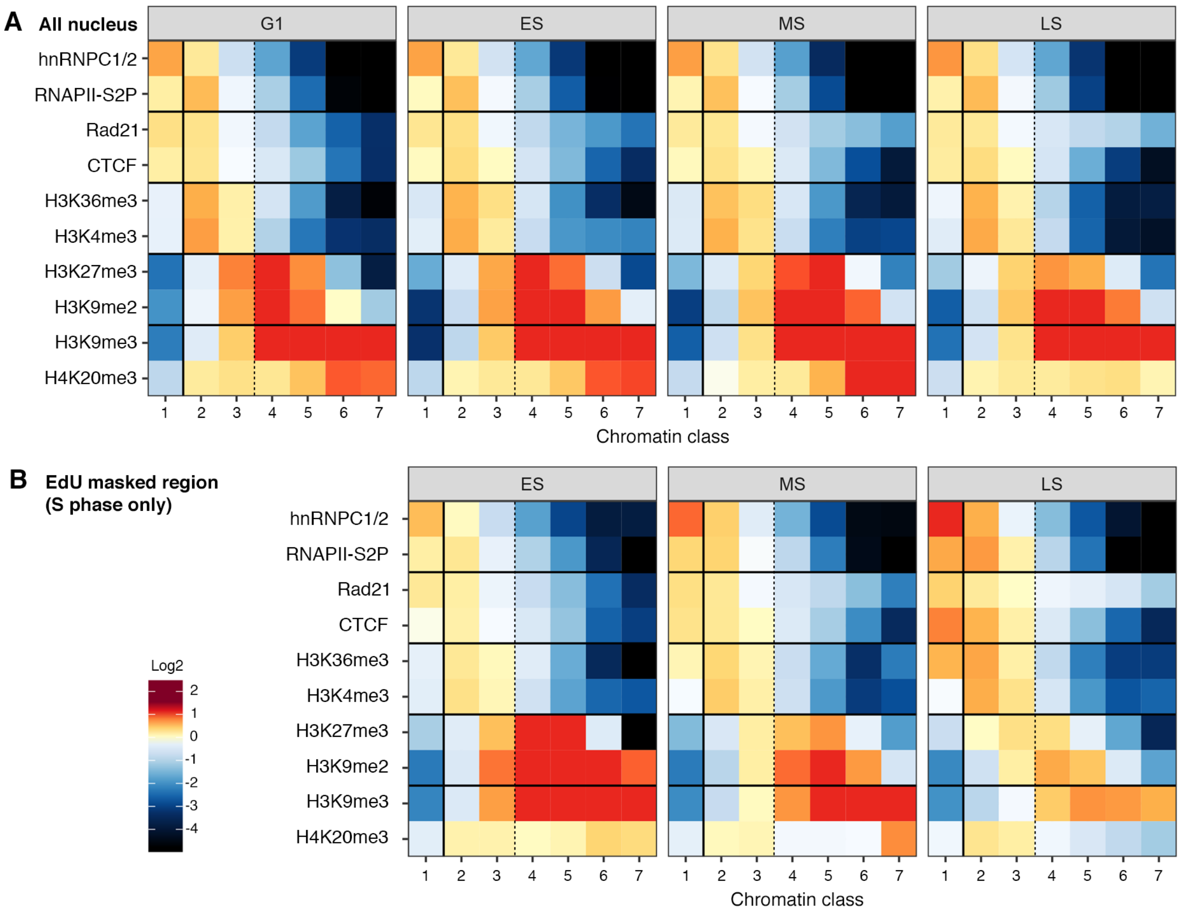

Fig. S7. Functional marker distribution in different S-phase stages of mouse C127 mammary epithelial cells. (**A**-**B**) Heatmaps of enrichment or depletion of IF signals relative to a random distribution (plotted in log2-fold change), per chromatin class, in the whole nuclear volume (A) or for a local EdU-submask (B). One heatmap per cell cycle stage. Number of cells: G1 =320, ES = 225, MS = 227, LS = 230.

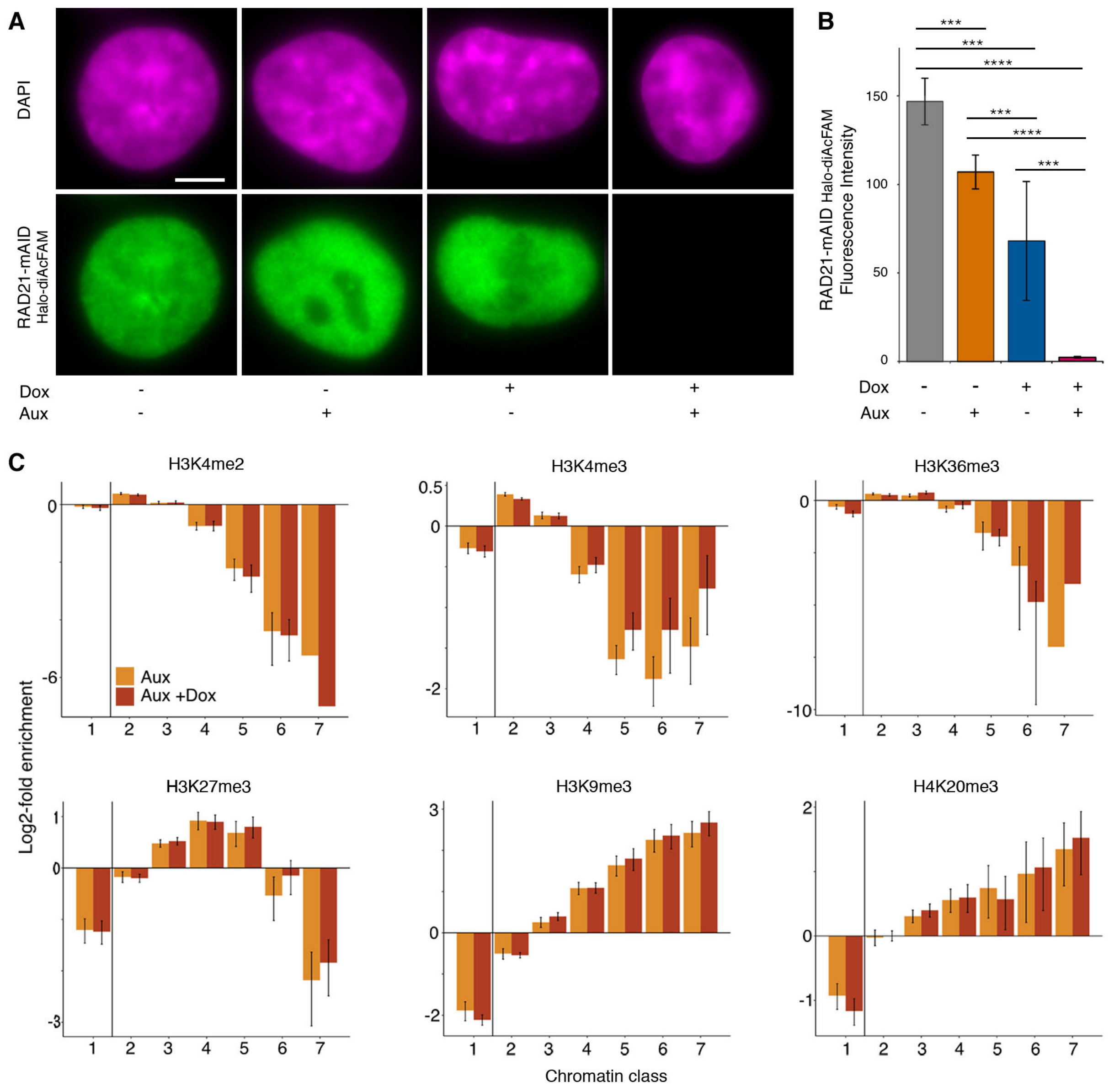

Fig. S8. Characterization of HCT116 Tet-OsTIR1 RAD21-mAID-Halo cell line and functional marker distribution for RAD21 ablation. (**A**) Representative widefield fluorescence microscopy images of DAPI and RAD21-mAID after mock induction, 2 h auxin (aux), 16 h doxycycline (dox), or both 2 h aux and 16 h dox (left to right respectively). Scale bar: 5 μm. (**B**) Quantification of the fluorescence intensity in the different conditions of (A). N = 10 cells per condition (error bars = SD). Significance values P: **** ≤ 0.0001; *** ≤ 0.001. (**C**) Bar plots of the over/under representation of each histone PTM analyzed in each of the segmented chromatin classes, with or without RAD21 ablation (6h aux +/- 16 h dox), in HCT116 cells. Plots show average log-2 value, error bars indicate 95% confidence interval. Numbers of cells for each marker is provided in Table S1.

Table S1. Average number of foci analyzed per marker.

	C127			
**Marker**	**G1** (*N*=433)	**ES** (*N*=225)	**MS** (*N*=227)	**LS** (*N*=230)
CTCF	7130 ± 2025 (40)	4784 ± 1699 (30)	4052 ± 2732 (30)	3878 ± 3360 (30)
H3K27me3	2590 ± 1500 (40)	1906 ± 1727 (20)	1940 ± 1734 (18)	1949 ± 1576 (20)
H3K36me2	3260 ± 1993 (20)	-	-	-
H3K36me3	1323 ± 515 (40)	2822 ± 1396 (30)	2541 ± 1153 (30)	392 ± 356 (30)
H3K4me2	2708 ± 1089 (20)	-	-	-
H3K4me3	5556 ± 1207 (40)	3692 ± 1777 (30)	2781 ± 2416 (29)	3104 ± 2778 (30)
H3K9me2	4787 ± 988 (30)	1169 ± 535 (15)	2753 ± 1405 (20)	2548 ± 1867 (20)
H3K9me3	1908 ± 1603 (30)	688 ± 230 (20)	809 ± 412 (20)	664 ± 229 (20)
H4K20me3	4523 ± 356 (20)	2598 ± 583 (20)	1959 ± 1196 (20)	2344 ± 392 (20)
H4K5ac	5177 ± 1527 (20)	-	-	-
hnRNPC1/2	4625 ± 781 (20)	1224 ± 423 (20)	865 ± 174 (20)	614 ± 220 (20)
Nascent RNA	2797 ± 1124 (13)	-	-	-
RAD21	1272 ± 391 (20)	981 ± 193 (10)	991 ± 102 (10)	1146 ± 244 (10)
RNAPII-S2P	5027 ± 1319 (40)	2830 ± 1860 (30)	2698 ± 2530 (30)	3019 ± 2360 (30)
SAF-A	4655 ± 1821 (20)	-	-	-
Smc3	615 ± 263 (20)	-	-	-

	HCT116 (RAD21-mAID)		HeLa
	**+ RAD21** (*N*=80) (6 h Auxin)	**- RAD21** (*N*=104) (16 h Dox + 6 h Auxin)	**G1** (*N*=41)
H3K27me3	1077 ± 516 (20)	1379 ± 407 (30)	-
H3K36me3	551 ± 138 (10)	790 ± 263 (12)	-
H3K4me2	2620 ± 413 (10)	3063 ± 412 (12)	-
H3K4me3	7409 ± 1252 (20)	8155 ± 1420 (30)	7341 ± 1785 (10)
H3K9me3	1182 ± 204 (10)	1771 ± 174 (10)	5837 ± 525 (11)
H4K20me3	2316 ± 387 (10)	2246 ± 272 (10)	-
hnRNPC1/2	-	-	7531 ± 1463 (10)
RNAPII-S2P	-	-	3719 ± 819 (10)

Average number of IF spots ± SD of each marker per cell for (*N*) number of cells in each condition, after high stringency filtering to avoid false positives. Cell cycle stages are abbreviated to ES, MS and LS for early, mid and late S phase. To maintain consistency, the same mammalian antibodies were used throughout this study, however the affinity of some antibodies is greater or decreased in either mouse (C127) or human (HCT116, HeLa) cells.

Table S2. Primary and secondary antibodies used in this study.

Antibodies	Source	Identifier
Primary antibodies:		
Rabbit mAb anti-hnRNP C1 + C2	Abcam	ab133607
Mouse mAb anti-Rad21	Merck Millipore	05-908
Rabbit pAb anti-SMC3	Bethyl Laboratories Inc.	A300-060A
Rabbit mAb anti-CTCF	Cell Signalling Tech.	D31H2
Rabbit pAb anti-SAF-A	Abcam	ab20666
Rabbit pAb anti-RNAPII ser2P	Abcam	ab5095
Rabbit pAb anti-H3K36me2	Active Motif	39255
Rabbit pAb anti-H3K36me3	Active Motif	61102
Mouse mAb anti-H3K4me2	Active Motif	39679
Rabbit pAb anti-H3K4me3	Active Motif	39159
Mouse mAb anti-H3K27me3	Abcam	ab6002
Rabbit pAb anti-H4K20me3	Abcam	ab9053
Mouse mAb anti-H3K9me2	Abcam	ab1220
Mouse mAb anti-H3K9me3	Active Motif	61013
Rabbit mAb anti-H4K5ac	Merck Millipore	04118
Primary antibodies (directly conjugated):		
Alexa Fluor 488 Rat mAb anti-BrdU	Abcam	ab220074
Secondary antibodies:		
Alexa Fluor 488 Goat pAb anti-Mouse-IgG	Thermo Fisher Scientific	A11029
Alexa Fluor 488 Goat pAb anti Rabbit-IgG	Thermo Fisher Scientific	A11034
Alexa Fluor 594 Donkey pAb anti-Mouse-IgG	Thermo Fisher Scientific	A21203
Alexa Fluor 594 Donkey pAb anti-Rabbit-IgG	Thermo Fisher Scientific	A21207

Movie S1. Super-resolution imaging of chromatin dynamics.
Live cell 3D-SIM of a human HeLa cell stably expressing histone H2B-GFP. Dataset corresponds to Figure 1D, recorded with time intervals of 2 s. For each frame, 7 z positions with a distance of 125 nm were acquired, totaling 105 raw images (5 phases, 3 angles, 7 z positions), covering a height of 0.75 μm. The reconstructed z-sections were maximum intensity projected, corrected for bleaching, and registered to compensate for cell motion and nuclear deformation. Note that high frequency noise ('hammer finish') artifacts become increasingly prominent with consecutive time points due to the reduction of signal-to-noise over time. For the movie an 'Orange Hot' LUT was chosen.

Movie S2. Super-resolution imaging of chromatin dynamics.
Live cell 3D-SIM of a human HeLa cell stably expressing histone H2B-GFP. Dataset corresponding to Figure S3A, recorded with time intervals of 3 s. Processing was performed as described for Movie S1.

Movie S3. Super-resolution imaging of chromatin dynamics.
Live cell 3D-SIM of a human HeLa cell stably expressing histone H2B-GFP. Dataset corresponding to Figure S3B, recorded with time intervals of 30 s. Processing was performed as described for Movie S1.

Movie S4. Quantitative analysis of chromatin dynamics.
Flow field analysis of the dataset shown in Movie S1, corresponding to Figure S3A, highlighting changes in motion direction between frames, characteristic for an elastic and coherent motion of chromatin features. Note that only changes for time lags of 1 frame are displayed, while the for subsequent correlation analyses (Fig. S3B) all time lags are included.

Movie S5. FIB-SEM sectioning through a HeLa cell nucleus.
Serial sections were taken perpendicular to the growth surface with a 4 nm isotropic voxel size. Movie shows a representative subvolume of the dataset presented in Figures 1E, F and Figure S3C-E. Data was downscaled to 8 nm voxel size and jpeg compressed to reduce file size.

Movie S6. 3D volume rendering of FIB-SEM imaged HeLa cell nucleus.
Movie shows subvolume of the dataset presented in Figures 1E, F and Figure S3C-E at 4 nm isotropic voxel resolution. Chromatin (magenta) and RNPs (green spots) were segmented using Ilastik software and rendered with Imaris.

REFERENCES AND NOTES

1. J. H. Gibcus, J. Dekker, The hierarchy of the 3D genome. *Mol. Cell* **49**, 773–782 (2013).

2. M. J. Rowley, V. G. Corces, Organizational principles of 3D genome architecture. *Nat. Rev. Genet.* **19**, 789–800 (2018).

3. K. Luger, A. W. Mäder, R. K. Richmond, D. F. Sargent, T. J. Richmond, Crystal structure of the nucleosome core particle at 2.8 Å resolution. *Nature* **389**, 251–260 (1997).

4. T. Cremer, C. Cremer, Chromosome territories, nuclear architecture and gene regulation in mammalian cells. *Nat. Rev. Genet.* **2**, 292–301 (2001).

5. C. Adriaens, L. A. Serebryannyy, M. Feric, A. Schibler, K. J. Meaburn, N. Kubben, P. Trzaskoma, S. Shachar, S. Vidak, E. H. Finn, V. Sood, G. Pegoraro, T. Misteli, Blank spots on the map: Some current questions on nuclear organization and genome architecture. *Histochem. Cell Biol.* **150**, 579–592 (2018).

6. J. R. Dixon, S. Selvaraj, F. Yue, A. Kim, Y. Li, Y. Shen, M. Hu, J. S. Liu, B. Ren, Topological domains in mammalian genomes identified by analysis of chromatin interactions. *Nature* **485**, 376–380 (2012).

7. A. D. Schmitt, M. Hu, B. Ren, Genome-wide mapping and analysis of chromosome architecture. *Nat. Rev. Mol. Cell Biol.* **17**, 743–755 (2016).

8. E. Lieberman-Aiden, N. L. van Berkum, L. Williams, M. Imakaev, T. Ragoczy, A. Telling, I. Amit, B. R. Lajoie, P. J. Sabo, M. O. Dorschner, R. Sandstrom, B. Bernstein, M. A. Bender, M. Groudine, A. Gnirke, J. Stamatoyannopoulos, L. A. Mirny, E. S. Lander, J. Dekker, Comprehensive mapping of long-range interactions reveals folding principles of the human genome. *Science* **326**, 289–293 (2009).

9. B. van Steensel, A. S. Belmont, Lamina-associated domains: Links with chromosome architecture, heterochromatin, and gene repression. *Cell* **169**, 780–791 (2017).

10. J. E. Phillips, V. G. Corces, CTCF: Master weaver of the genome. *Cell* **137**, 1194–1211 (2009).

11. D. G. Lupiáñez, K. Kraft, V. Heinrich, P. Krawitz, F. Brancati, E. Klopocki, D. Horn, H. Kayserili, J. M. Opitz, R. Laxova, F. Santos-Simarro, B. Gilbert-Dussardier, L. Wittler, M. Borschiwer, S. A. Haas, M. Osterwalder, M. Franke, B. Timmermann, J. Hecht, M. Spielmann, A. Visel, S. Mundlos, Disruptions of topological chromatin domains cause pathogenic rewiring of gene-enhancer interactions. *Cell* **161**, 1012–1025 (2015).

12. M. Merkenschlager, E. P. Nora, CTCF and cohesin in genome folding and transcriptional gene regulation. *Annu. Rev. Genomics Hum. Genet.* **17**, 17–43 (2016).

13. S. Yatskevich, J. Rhodes, K. Nasmyth, Organization of chromosomal DNA by SMC complexes. *Annu. Rev. Genet.* **53**, 445–482 (2019).

14. S. S. P. Rao, S.-C. Huang, B. G. St. Hilaire, J. M. Engreitz, E. M. Perez, K.-R. Kieffer-Kwon, A. L. Sanborn, S. E. Johnstone, G. D. Bascom, I. D. Bochkov, X. Huang, M. S. Shamim, J. Shin, D. Turner, Z. Ye, A. D. Omer, J. T. Robinson, T. Schlick, B. E. Bernstein, R. Casellas, E. S. Lander, E. L. Aiden, Cohesin loss eliminates all loop domains. *Cell* **171**, 305–320.e24 (2017).

15. T. Nagano, Y. Lubling, T. J. Stevens, S. Schoenfelder, E. Yaffe, W. Dean, E. D. Laue, A. Tanay, P. Fraser, Single-cell Hi-C reveals cell-to-cell variability in chromosome structure. *Nature* **502**, 59–64 (2013).

16. I. M. Flyamer, J. Gassler, M. Imakaev, H. B. Brandão, S. V. Ulianov, N. Abdennur, S. V. Razin, L. A. Mirny, K. Tachibana-Konwalski, Single-nucleus Hi-C reveals unique chromatin reorganization at oocyte-to-zygote transition. *Nature* **544**, 110–114 (2017).

17. B. Bintu, L. J. Mateo, J.-H. Su, N. A. Sinnott-Armstrong, M. Parker, S. Kinrot, K. Yamaya, A. N. Boettiger, X. Zhuang, Super-resolution chromatin tracing reveals domains and cooperative interactions in single cells. *Science* **362**, eaau1783 (2018).

18. E. H. Finn, G. Pegoraro, H. B. Brandão, A.-L. Valton, M. E. Oomen, J. Dekker, L. Mirny, T. Misteli, Extensive Heterogeneity and intrinsic variation in spatial genome organization. *Cell* **176**, 1502–1515.e10 (2019).

19. E. P. Nora, B. R. Lajoie, E. G. Schulz, L. Giorgetti, I. Okamoto, N. Servant, T. Piolot, N. L. van

Berkum, J. Meisig, J. Sedat, J. Gribnau, E. Barillot, N. Blüthgen, J. Dekker, E. Heard, Spatial partitioning of the regulatory landscape of the X-inactivation centre. *Nature* **485**, 381–385 (2012).

20. K. Maeshima, R. Rogge, S. Tamura, Y. Joti, T. Hikima, H. Szerlong, C. Krause, J. Herman, E. Seidel, J. De Luca, T. Ishikawa, J. C. Hansen, Nucleosomal arrays self-assemble into supramolecular globular structures lacking 30-nm fibers. *EMBO J.* **35**, 1115–1132 (2016).

21. T. Nozaki, R. Imai, M. Tanbo, R. Nagashima, S. Tamura, T. Tani, Y. Joti, M. Tomita, K. Hibino, M. T. Kanemaki, K. S. Wendt, Y. Okada, T. Nagai, K. Maeshima, Dynamic organization of chromatin domains revealed by super-resolution live-cell imaging. *Mol. Cell* **67**, 282–293.e7 (2017).

22. A. N. Boettiger, B. Bintu, J. R. Moffitt, S. Wang, B. J. Beliveau, G. Fudenberg, M. Imakaev, L. A. Mirny, C.-t. Wu, X. Zhuang, Super-resolution imaging reveals distinct chromatin folding for different epigenetic states. *Nature* **529**, 418–422 (2016).

23. K. Maeshima, K. Kaizu, S. Tamura, T. Nozaki, T. Kokubo, K. Takahashi, The physical size of transcription factors is key to transcriptional regulation in chromatin domains. *J. Phys. Condens. Matter* **27**, 064116 (2015).

24. Q. Szabo, D. Jost, J.-M. Chang, D. I. Cattoni, G. L. Papadopoulos, B. Bonev, T. Sexton, J. Gurgo, C. Jacquier, M. Nollmann, F. Bantignies, G. Cavalli, TADs are 3D structural units of higher-order chromosome organization in *Drosophila*. *Sci. Adv.* **4**, eaar8082 (2018).

25. L. Schermelleh, A. Ferrand, T. Huser, C. Eggeling, M. Sauer, O. Biehlmaier, G. P. C. Drummen, Super-resolution microscopy demystified. *Nat. Cell Biol.* **21**, 72–84 (2019).

26. L. Schermelleh, P. M. Carlton, S. Haase, L. Shao, L. Winoto, P. Kner, B. Burke, M. C. Cardoso, D. A. Agard, M. G. L. Gustafsson, H. Leonhardt, J. W. Sedat, Subdiffraction multicolor imaging of the nuclear periphery with 3D structured illumination microscopy. *Science* **320**, 1332–1336 (2008).

27. Y. Markaki, M. Gunkel, L. Schermelleh, S. Beichmanis, J. Neumann, M. Heidemann, H. Leonhardt, D. Eick, C. Cremer, T. Cremer, Functional nuclear organization of transcription and DNA replication: A topographical marriage between chromatin domains and the interchromatin compartment. *Cold Spring Harb. Symp. Quant. Biol.* **75**, 475–492 (2010).

28. D. Smeets, Y. Markaki, V. J. Schmid, F. Kraus, A. Tattermusch, A. Cerase, M. Sterr, S. Fiedler, J. Demmerle, J. Popken, H. Leonhardt, N. Brockdorff, T. Cremer, L. Schermelleh, M. Cremer, Three-dimensional super-resolution microscopy of the inactive X chromosome territory reveals a collapse of its active nuclear compartment harboring distinct Xist RNA foci. *Epigenetics Chromatin* **7**, 8 (2014).

29. H. A. Shaban, R. Barth, K. Bystricky, Formation of correlated chromatin domains at nanoscale dynamic resolution during transcription. *Nucleic Acids Res.* **46**, e77 (2018).

30. R. Bruinsma, A. Y. Grosberg, Y. Rabin, A. Zidovska, Chromatin hydrodynamics. *Biophys. J.* **106**, 1871–1881 (2014).

31. C. Y. Jao, A. Salic, Exploring RNA transcription and turnover *in vivo* by using click chemistry. *Proc. Natl. Acad. Sci. U.S.A.* **105**, 15779–15784 (2008).

32. C. S. Xu, K. J. Hayworth, Z. Lu, P. Grob, A. M. Hassan, J. G. García-Cerdán, K. K. Niyogi, E. Nogales, R. J. Weinberg, H. F. Hess, Enhanced FIB-SEM systems for large-volume 3D imaging. *eLife* **6**, ae25916 (2017).

33. C.-L. Chang, A. V. Weigel, M. S. Ioannou, H. A. Pasolli, C. S. Xu, D. R. Peale, G. Shtengel, M. Freeman, H. F. Hess, C. Blackstone, J. Lippincott-Schwartz, Spastin tethers lipid droplets to peroxisomes and directs fatty acid trafficking through ESCRT-III. *J. Cell Biol.* **218**, 2583–2599 (2019).

34. D. P. Hoffman, G. Shtengel, C. S. Xu, K. R. Campbell, M. Freeman, L. Wang, D. E. Milkie, H. A. Pasolli, N. Iyer, J. A. Bogovic, D. R. Stabley, A. Shirinifard, S. Pang, D. Peale, K. Schaefer, W. Pomp, C.-L. Chang, J. Lippincott-Schwartz, T. Kirchhausen, D. J. Solecki, E. Betzig, H. F. Hess, Correlative three-dimensional super-resolution and block-face electron microscopy of whole vitreously frozen cells. *Science* **367**, eaaz5357 (2020).

35. H. D. Ou, S. Phan, T. J. Deerinck, A. Thor, M. H. Ellisman, C. C. O'Shea, ChromEMT: Visualizing 3D chromatin structure and compaction in interphase and mitotic cells. *Science* **357**, eaag0025 (2017).

36. S. Berg, D. Kutra, T. Kroeger, C. N. Straehle, B. X. Kausler, C. Haubold, M. Schiegg, J. Ales, T. Beier, M. Rudy, K. Eren, J. I. Cervantes, B. Xu, F. Beuttenmueller, A. Wolny, C. Zhang, U. Koethe, F. A. Hamprecht, A. Kreshuk, Ilastik: Interactive machine learning for (bio)image analysis. *Nat. Methods* **16**, 1226–1232 (2019).

37. Y. Markaki, D. Smeets, S. Fiedler, V. J. Schmid, L. Schermelleh, T. Cremer, M. Cremer, The potential of 3D-FISH and super-resolution structured illumination microscopy for studies of 3D nuclear architecture: 3D structured illumination microscopy of defined chromosomal structures visualized by 3D (immuno)-FISH opens new perspectives for studies of nuclear architecture. *Bioessays* **34**, 412–426 (2012).

38. J. M. Brown, N. A. Roberts, B. Graham, D. Waithe, C. Lagerholm, J. M. Telenius, S. De Ornellas, A. M. Oudelaar, C. Scott, I. Szczerbal, C. Babbs, M. T. Kassouf, J. R. Hughes, D. R. Higgs, V. J. Buckle, A tissue-specific self-interacting chromatin domain forms independently of enhancer-promoter interactions. *Nat. Commun.* **9**, 3849 (2018).

39. F. Ochs, G. Karemore, E. Miron, J. Brown, H. Sedlackova, M.-B. Rask, M. Lampe, V. Buckle, L. Schermelleh, J. Lukas, C. Lukas, Stabilization of chromatin topology safeguards genome integrity. *Nature* **574**, 571–574 (2019).

40. S. Fakan, R. van Driel, The perichromatin region: A functional compartment in the nucleus that determines large-scale chromatin folding. *Semin. Cell Dev. Biol.* **18**, 676–681 (2007).

41. M. Cremer, K. Brandstetter, A. Maiser, S. S. P. Rao, V. Schmid, N. Mitra, S. Mamberti, K.-N. Klein, D. M. Gilbert, H. Leonhardt, M. C. Cardoso, E. L. Aiden, H. Harz, T. Cremer, Cohesin depleted cells pass through mitosis and reconstitute a functional nuclear architecture. *bioRxiv*, 816611 (2019).

42. R.-S. Nozawa, L. Boteva, D. C. Soares, C. Naughton, A. R. Dun, A. Buckle, B. Ramsahoye, P. C. Bruton, R. S. Saleeb, M. Arnedo, B. Hill, R. R. Duncan, S. K. Maciver, N. Gilbert, SAF-A regulates interphase chromosome structure through oligomerization with chromatin-associated RNAs. *Cell* **169**, 1214–1227.e18 (2017).

43. R. Zhao, T. Nakamura, Y. Fu, Z. Lazar, D. L. Spector, Gene bookmarking accelerates the kinetics of

post-mitotic transcriptional re-activation. *Nat. Cell Biol.* **13**, 1295–1304 (2011).

44. S. Dambacher, M. Hahn, G. Schotta, Epigenetic regulation of development by histone lysine methylation. *Heredity* **105**, 24–37 (2010).

45. G. Schotta, M. Lachner, K. Sarma, A. Ebert, R. Sengupta, G. Reuter, D. Reinberg, T. Jenuwein, A silencing pathway to induce H3-K9 and H4-K20 trimethylation at constitutive heterochromatin. *Genes Dev.* **18**, 1251–1262 (2004).

46. V. O. Chagin, C. S. Casas-Delucchi, M. Reinhart, L. Schermelleh, Y. Markaki, A. Maiser, J. J. Bolius, A. Bensimon, M. Fillies, P. Domaing, Y. M. Rozanov, H. Leonhardt, M. C. Cardoso, 4D Visualization of replication foci in mammalian cells corresponding to individual replicons. *Nat. Commun.* **7**, 11231 (2016).

47. V. O. Chagin, B. Reinhart, A. Becker, O. Mortusewicz, K. L. Jost, A. Rapp, H. Leonhardt, M. C. Cardoso, Processive DNA synthesis is associated with localized decompaction of constitutive heterochromatin at the sites of DNA replication and repair. *Nucleus* **10**, 231–253 (2019).

48. I. F. Davidson, B. Bauer, D. Goetz, W. Tang, G. Wutz, J.-M. Peters, DNA loop extrusion by human cohesin. *Science* **366**, 1338–1345 (2019).

49. T. Natsume, T. Kiyomitsu, Y. Saga, M. T. Kanemaki, Rapid protein depletion in human cells by auxin-inducible degron tagging with short homology donors. *Cell Rep.* **15**, 210–218 (2016).

50. J. Kind, L. Pagie, H. Ortabozkoyun, S. Boyle, S. S. de Vries, H. Janssen, M. Amendola, L. D. Nolen, W. A. Bickmore, B. van Steensel, Single-cell dynamics of genome-nuclear lamina interactions. *Cell* **153**, 178–192 (2013).

51. I. Solovei, M. Kreysing, C. Lanctôt, S. Kösem, L. Peichl, T. Cremer, J. Guck, B. Joffe, Nuclear architecture of rod photoreceptor cells adapts to vision in mammalian evolution. *Cell* **137**, 356–368 (2009).

52. B. Joffe, H. Leonhardt, I. Solovei, Differentiation and large scale spatial organization of the genome. *Curr. Opin. Genet. Dev.* **20**, 562–569 (2010).

53. T. Cremer, M. Cremer, B. Hübner, H. Strickfaden, D. Smeets, J. Popken, M. Sterr, Y. Markaki, K. Rippe, C. Cremer, The 4D nucleome: Evidence for a dynamic nuclear landscape based on co-aligned active and inactive nuclear compartments. *FEBS Lett.* **589**, 2931–2943 (2015).

54. A. F. Palazzo, E. S. Lee, Non-coding RNA: What is functional and what is junk? *Front. Genet.* **6**, 2 (2015).

55. A. I. Järvelin, M. Noerenberg, I. Davis, A. Castello, The new (dis)order in RNA regulation. *Cell Commun. Signal* **14**, 9 (2016).

56. Y. Shin, Y.-C. Chang, D. S. W. Lee, J. Berry, D. W. Sanders, P. Ronceray, N. S. Wingreen, M. Haataja, C. P. Brangwynne, Liquid nuclear condensates mechanically sense and restructure the genome. *Cell* **175**, 1481–1491.e13 (2018).

57. W. Xiang, M. J. Roberti, J.-K. Hériché, S. Huet, S. Alexander, J. Ellenberg, Correlative live and super-resolution imaging reveals the dynamic structure of replication domains. *J. Cell Biol.* **217**, 1973–1984 (2018).

58. J. Rhodes, D. Mazza, K. Nasmyth, S. Uphoff, Scc2/Nipbl hops between chromosomal cohesin rings after loading. *eLife* **6**, e30000 (2017).

59. E. Miron, C. Innocent, S. Heyde, L. Schermelleh, in *Methods in Molecular Biology: Chromosome Architecture: Methods and Protocols*, M. C. Leake, Ed. (Springer, 2016), vol. 1431, pp. 127–140.

60. F. Kraus, E. Miron, J. Demmerle, T. Chitiashvili, A. Budco, Q. Alle, A. Matsuda, H. Leonhardt, L. Schermelleh, Y. Markaki, Quantitative 3D structured illumination microscopy of nuclear structures. *Nat. Protoc.* **12**, 1011–1028 (2017).

61. J. Demmerle, C. Innocent, A. J. North, G. Ball, M. Müller, E. Miron, A. Matsuda, I. M. Dobbie, Y. Markaki, L. Schermelleh, Strategic and practical guidelines for successful structured illumination microscopy. *Nat. Protoc.* **12**, 988–1010 (2017).

62. G. Ball, J. Demmerle, R. Kaufmann, I. Davis, I. M. Dobbie, L. Schermelleh, SIMcheck: A toolbox for successful super-resolution structured illumination microscopy. *Sci. Rep.* **5**, 15915 (2015).

63. A. Matsuda, L. Schermelleh, Y. Hirano, T. Haraguchi, Y. Hiraoka, Accurate and fiducial-marker-free correction for three-dimensional chromatic shift in biological fluorescence microscopy. *Sci. Rep.* **8**, 7583 (2018).

64. J. Schindelin, I. Arganda-Carreras, E. Frise, V. Kaynig, M. Longair, T. Pietzsch, S. Preibisch, C. Rueden, S. Saalfeld, B. Schmid, J.-Y. Tinevez, D. J. White, V. Hartenstein, K. Eliceiri, P. Tomancak, A. Cardona, Fiji: An open-source platform for biological-image analysis. *Nat. Methods* **9**, 676–682 (2012).

65. V. J. Schmid, M. Cremer, T. Cremer, Quantitative analyses of the 3D nuclear landscape recorded with super-resolved fluorescence microscopy. *Methods* **123**, 33–46 (2017).

66. H. A. Shaban, R. Barth, K. Bystricky, Hi-D: Nanoscale mapping of nuclear dynamics in single living cells. *Genome Biol.* **21**, 95 (2020).

67. B. Bonev, N. M. Cohen, Q. Szabo, L. Fritsch, G. L. Papadopoulos, Y. Lubling, X. Xu, X. Lv, J.-P. Hugnot, A. Tanay, G. Cavalli, Multiscale 3D genome rewiring during mouse neural development. *Cell* **171**, 557–572.e24 (2017).
